# Effective Removal of Acid Dye in Synthetic and Silk Dyeing Effluent: Isotherm and Kinetic Studies

**DOI:** 10.1021/acsomega.1c04111

**Published:** 2021-12-30

**Authors:** Elizaveta Sterenzon, Vinod Kumar Vadivel, Yoram Gerchman, Thomas Luxbacher, Ramsundram Narayanan, Hadas Mamane

**Affiliations:** † School of Mechanical Engineering, Faculty of Engineering, Tel Aviv University, Tel Aviv 69978, Israel; ‡ Department of Biology and Environment, Faculty of Natural Science, University of Haifa and Oranim College, Tivon 3600600, Israel; § Anton Paar GmbH, Anton Paar Str. 20, 8054 Graz, Austria; ∥ Faculty of Chemistry and Chemical Engineering, University of Maribor, 2000 Maribor, Slovenia; ⊥ Department of Civil Engineering, Kumaraguru College of Technology, Coimbatore, Tamil Nadu 641049, India

## Abstract

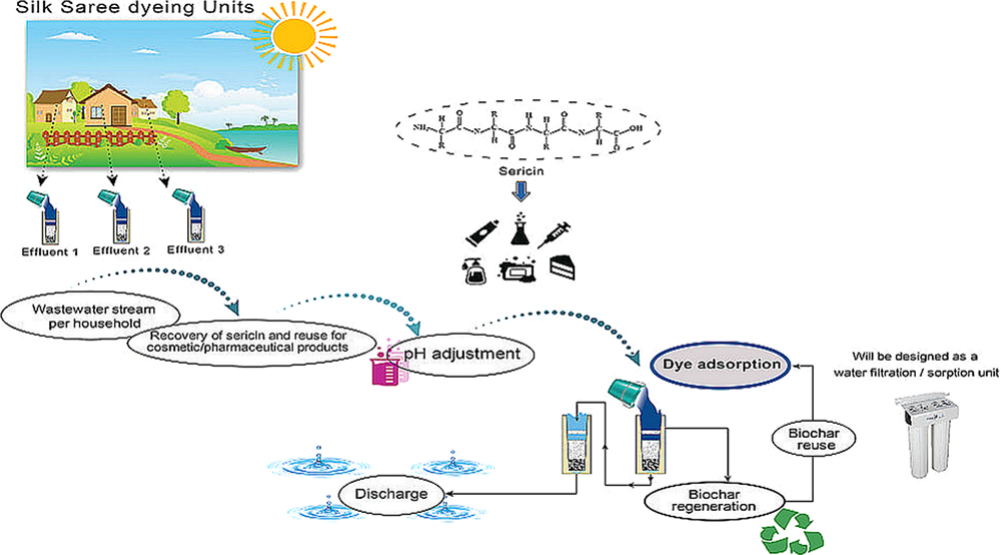

Here, we propose a low-cost, sustainable, and viable adsorbent (pine tree-derived biochar) to remove acid dyes such as acid violet 17 (AV), which is used in the silk dyeing industry. As a case study, the AV removal process was demonstrated using synthetic effluent and further as a proof of concept using real dye effluent produced from the Sirumugai textile unit in India. The pine tree-derived biochar was selected for removal of aqueous AV dye in batch and fixed-bed column studies. The adsorbent material was characterized for crystallinity (XRD), surface area (BET), surface morphology and elemental compositions (SEM–EDX), thermal stability (TGA), weight loss (DGA), and functional groups (FTIR). Batch sorption studies were performed to evaluate (i) adsorption at various pH values (at pH 2 to 7), (ii) isotherms (at 10, 25, and 35 °C) to assess the temperature effect on the sorption efficiency, and (iii) kinetics to reveal the effect of time, adsorbent dose, and initial concentration on the reaction rate. After systematic evaluation, 2 g/L biochar, 25 mg/L AV, pH 3, 40 °C, and 40 and 360 min in a completely mixed batch study resulted in 50 and 90% dye removal, respectively. The isoelectric point at pH 3.7 ± 0.2 results in maximum dye removal, therefore suggesting that monitoring the ratio of different effluent (acid/wash/dye) can improve the colorant removal efficiency. The Langmuir isotherm best fits with the sorption of AV to biochar, provided a maximal dye uptake of 29 mg/g at 40 °C, showing that adsorption was endothermic. Fixed-bed studies were conducted at room temperature with an initial dye concentration of 25 and 50 mg/L. The glass columns were packed with biochar (bed depth 20 cm, pore volume = 14 mL) at an initial pH of 5.0 and a 10 mL/min flow rate for 120 min. Finally, the regeneration of the adsorbent was achieved using desorption studies conducted under the proposed experimental conditions resulted in 90–93% removal of AV even after five cycles of regeneration.

## 1 Introduction

The demand for water in the industrial sector is expected to increase ∼300% during the first half of the 21st century.^1^ The textile industry is one of the largest water consumers in many developing countries, especially in Asia, second to agriculture.^2^ Therefore, this industry requires a large amount of water for the production process and is also one of the major producers of toxic and polluted wastewater.^3^ Over 7 × 10^7^ tons of synthetic dyes are produced yearly, and 10–15% are not integrated into the final product.^4^

Acid dyes are commonly used in the textile industry to dye protein fibers like silk, wool, angora, and synthetic nylon.^4^ Acid dyes are sodium or ammonium salts of carboxylic, phenolic, or sulphonic organic acids, which are highly soluble in water, and their dye molecules are negatively charged. Different technologies were applied to remove acid dyes, comprising electrocoagulation,^5^ biological,^6^ and physicochemical^7^ processes. Adsorption by porous materials is one of the most promising and affordable techniques for removing dissolved pollutants, serving as an alternative to energy-intensive technologies.^8^

Activated carbon and lignin-based hydrogels are widely used adsorbents.^9−11^ However, biochar is inexpensive, abundant, has comparable adsorption capacity, and can be used as an affordable alternative.^12^ Low-temperature pyrolysis of biomass rich with carbon in low-oxygen conditions forms biochar.^13^ The porosity and high surface area of biochar make it an excellent adsorbent of organic contaminants and heavy metals from wastewater.^12^ These advances have led to a growing interest in using biochar for water treatment. However, most of the research of today focuses on its abilities in soil fertilization.^14^

Adsorption of textile dyes was examined with various biochars, dyes, and process parameters such as temperature, pH, and agitation time.^15^ Most studies focused on removing pure, highly concentrated dye solutions that do not represent the actual effluent from domestic real dye houses. Real effluent may contain lower dye concentration and additional substances such as salts, detergents, solids, and fiber residuals, significantly affecting sorption capacity. This study focused on developing a process for removing synthetic and real acid dyes from aqueous solutions and designing a treatment process. We also examined the removal efficiency of AV-17 in the presence of salt solution (NaCl) to simulate real dye effluent. Moreover, dye removal was demonstrated on real nonmixed and mixed effluents from textile dye houses located at Coimbatore Tamil Nadu, South India.

The Indian textile industry counts among the leading textile industries worldwide according to the IBEF (India Brand Equity Foundation).^16^ The Indian textile and apparel industry contributed 2% to the GDP of the country. The textile industry is the second largest after agriculture in India, employing over 45 million people directly and 60 million indirectly.^17^ It contributes about 14% of the total industrial production of India. This article aims to provide a sustainable solution for the real textile effluent from the house dyers at Coimbatore, Tamil Nadu, India. Their main products are soft silk and Kovai Kora traditional sarees.

## 2 Materials and Methods

### 2.1 Characteristics of Pinewood Biochar

Pine biochar was obtained from EcoAeonAgro. The biochar was used by drying at 105 °C and sieved in a sieving machine (450 W, ALS Ltd., Israel). The particle size distribution of the biochar was in the ranges of ≥1000 μm, 55.65%; 500–1000 μm, 28.02%; and <500 μm, 16.33%. X-ray diffraction (XRD) with Cu K_α_ radiation (Bruker D8 Advance) measured the phase and crystallinity. Thermal stability and weight loss were studied using thermogravimetric analysis (TGA) according to the DIN 51719 method using a high-sensitivity thermogravimetric analyzer (Q5000 TGA-IR, TA Instruments) operating from room temperature to 550 °C at a heating rate of 5 °C min^–1^. Brunauer–Emmet–Teller (BET) surface area, pore-volume, and radius were determined by N_2_ adsorption–desorption isotherms collected at 77 K using a Quantachrome instrument (Q5000 TGA-IR, TA Instruments). Fourier transform infrared (FTIR) spectroscopy (Tensor 27-IR, Bruker, USA) determines functional groups on the biochar. The surface structure and morphology of the biochar were observed by QUANTA 200 FEG Environmental Scanning Electron Microscope (ESEM) with afield-emission gun electron source. The zeta potential of the biochar was determined from streaming potential measurements using an electrokinetic analyzer (SurPASS3, Anton Paar GmbH, Austria). The zeta potential was calculated from streaming potentials using the Helmholtz–Smoluchowski equation^18^
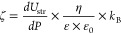
1where *ζ* (mV) is the zeta potential, *dU*_str_ (mV) is the streaming potential, *dP* (mbar) is the pressure gradient, η (mPa s) is the viscosity of water, ε and ε_0_ (8.854 × 10^–12^ A^2^ s^4^/kg·m^3^), the dielectric coefficient of water and the permittivity in free space, respectively, and *k*_B_ (mS/m) the conductivity of the aqueous solution. One hundred fifty milligrams of biochar were suspended in deionized water and transferred into the cylindrical cell of the SurPASS 3 instrument. The biochar sample was covered on both sides by filter disks with 25 μm mesh. An aqueous 0.001 mol/L KCl solution was used for the streaming potential measurement. First, the biochar sample plug was rinsed with 400 mL of deionized water and 80 mL of 0.001 mol/L KCl, which was discarded. The biochar sample plug was compressed gently to provide a permeability toward the liquid flow of 122 ± 6 (permeability index provided by SurPASS 3 software), which was maintained during the series of measurements.

### 2.2 Textile Dye

Acid Violet 17 (AV), an anionic dye dark violet in color, was provided by Colourtex Industries Ltd., India, and has a molecular formula of C_41_H_44_N_3_NaO_6_S_2_ (Figure S1). The AV (500 mg/L) stock solution was prepared and subsequently diluted with DDW to get the required dye concentration for each experiment. However, to simulate real dye wastewater, the conductivity of the dye solution was adjusted with NaCl to be ∼36 mS/cm for all the experiments. Due to their chemical structures, the concentration of anionic dyes in aqueous solutions can be determined by spectroscopic measurements in the visible spectrum of light. The maximum absorbance wavelength value of the dye solutions was 540 nm (Spark 10 M plate reader, Tecan, Switzerland).

The dye uptake, *q_t_*(mg/g), which is the amount of the adsorbed dye at time *t*( min ) on a specific amount of biochar, was determined using the following equation
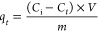
2Where *C*_i_ and *C_t_*(mg/g) are the initial AV concentration and the concentration at time *t*, respectively, *V*(L) is the volume of the dye solution, and *m*(g) is the biochar dose. The dye removal percentage was calculated using the following equation:
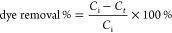
3

### 2.3 AV-17 Removal in a Batch Reactor

Batch experiments were conducted to study AV removal efficiency and the effects of different variables on adsorption as initial solution pH, biochar dose, and initial dye concentration. Fifty milliliters of AV solution was inserted into a 100 mL conical glass flask at the desired concentration. A known amount of biochar was added to the solution and agitated at 25 °C in the mechanical shaker incubator (061450100 CTS-100B) at 150 rpm. The supernatant was separated using centrifugation (14,000 rpm for 10 min), and dye removal efficiency was determined using a UV spectrophotometer. The effect of pH on removing AV dye was studied over the pH range of 3 to 9 with 2 g/L of biochar. Initial pH was adjusted with 1 M HCl and 1 M NaOH solutions. The effect of biochar dose was determined by agitating 0.5, 1, 2, 3, and 4 g/L of biochar with 25 or 50 mg/L of AV solution.

### 2.4 Adsorption Kinetics

Kinetic studies were carried out using a mechanical shaker at 25 °C and 150 rpm. Adsorbent (2 g/L) was agitated with 25 and 50 mg/L of AV solution; afterward, samples were withdrawn at predetermined time intervals from 5 to 480 min. Three linear forms of kinetic models were examined. The first model is the pseudo-first-order equation of Lagergren for the sorption of a liquid/solid system based on the solid capacity, which is the most widely used expression for liquid-phase sorption processes and can be represented as^9−11,19^
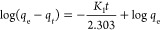
4where *q*_e_(mg/g),is the amount of dye adsorbed per unit mass of biochar at equilibrium and *K*_1_(min^–1^) is the rate constant of the pseudo-first-order model.

The second model is the Lagergren pseudo-second-order kinetics and is generally employed in the form proposed by Ho and McKay^20^
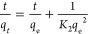
5where, K_2_(g/mg min ) is the rate of the pseudo-second-order model. The initial rate of biosorption, *h*(mg/g min ), is defined to be
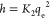
6

The third model is the intraparticle diffusion model that corresponds to a diffusion-controlled process, with the amount of the adsorbed dye, *q_t_*(mg/g), at time *t* described by
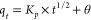
7

where *K*_p_(mg/g min^1/2^) is the intraparticle diffusion rate constant and θ(mg/g) is the constant that is proportional to the thickness of the boundary layer; the larger the value of θ, the greater the boundary layer thickness. The values of θ and *K*_p_ indicate the controlling mechanisms of the adsorption.^21^

### 2.5 Temperature and Concentration Effect

Different initial concentrations of AV were used; 10, 25, 40, 50, 65, 80, and 100 mg/L. A mass of 0.1 g of biochar was agitated with 50 mL of AV solution (2 g/L) at pH 3 for 8 h at different temperatures (25–50 °C) in a mechanical shaker at 150 rpm. Langmuir and Freundlich’s adsorption isotherms were used to analyze the obtained experimental data.

According to Langmuir, adsorption is a monolayer process, which assumes that a limited number of adsorption sites exist on the adsorbent surface. Once a dye molecule captures a sorption site, no further adsorption can occur at that site because it can hold at most one molecule of adsorbate.^22^ The mathematical expression is
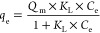
8where *Q*_m_ (mg/g) is the maximum monolayer adsorption capacity of the biochar, *C*_e_ (mg/L) is the concentration of adsorbate in the solution at equilibrium, and *K*_L_ (L/mg) is the Langmuir constant that describes the affinity of adsorbate to the biochar. Therefore, to plot the Langmuir isotherm, the linear expression will be used:
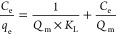
9When the Langmuir model fits the experimental data well, a dimensionless constant separation factor, *R*_L_, can be calculated:
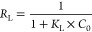
10

Here, *C*_0_ (mg/L) is the highest initial dye concentration. This factor, *R*_L_, indicates the shape of the isotherm and the adsorption nature: *R*_L_ = 0, 0 < *R*_L_ < 1, *R*_L_ = l, and *R_L_* > l represent irreversible, favorable, linear, and unfavorable adsorption, respectively.^23^ The Freundlich isotherm is an empirical equation that describes heterogeneous systems. It assumes adsorption occurs on a heterogeneous surface by a multilayer adsorption mechanism and that the adsorption is increasing with the increase of the contaminant concentration according to
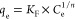
11

where *K*_F_ (mg/g)(L/mg)1/*n* is the Freundlich sorption coefficient, which indicates that the adsorption capacity and 1/*n* are dimensionless Freundlich adsorption intensity parameters. These parameters can be determined by the linear form of the Freundlich expression that can be obtained by taking logarithms of the nonlinear form^9−11,24^
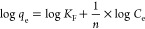
12

### 2.6 Thermodynamic Studies

The thermodynamic parameters of the adsorption were determined using the following equations
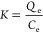
13
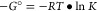
14
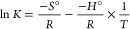
15where −*G*^°^ is the free energy, −*H*^°^ is the enthalpy, −*S*^°^ is the entropy of the process, *R* is the gas constant (*R* = 8.314 J/mol K), *K* is the equilibrium constant, and *T* is the operating temperature in Kelvin. −*H*^°^ and −*S*^°^ were calculated from the slope and intercept of the plots of ln*K* versus 1/*T*.

### 2.7 Regeneration

More environmental and economic regeneration studies for biochar are considered a suitable approach toward water treatment processes. Three regeneration fluids have been tested in the preliminary studies: absolute ethanol, 70% ethanol, and 0.1 M NaOH. The absolute ethanol observed the best dye desorption results; hence, it was chosen for further regeneration studies. Biochar (0.1 g) was agitated with 50 mL of AV solution at a concentration of 50 mg/L for 40 min at 150 rpm and 25 °C. After 40 min, the solution was removed by a syringe and replaced with 10 mL of absolute ethanol. The biochar was sonicated (MRC, Ultrasonic bath, power 100 watts/ 40 kHz) for 5 min; afterward, the ethanol was removed and the regenerated biochar was dried at 105 °C for 1 h. The dry biochar was left to cool to room temperature in the desiccator for 1 h, and then its weight was measured to estimate the mass loss. The regenerated biochar was again tested for dye adsorption as was described previously. The regeneration was done for five cycles.

### 2.8 Removal of AV in Fixed-Bed Column Experiments

Column adsorption tests were conducted to determine the removal of AV from water solutions by biochar under continuous flow. The experiments were performed with dye concentrations of 25 and 50 mg/L AV solution, and the dye solution flow rate was set to be constant at 10 mL/min. Fixed-bed column experiments were conducted using 11 mm inner diameter and 60 cm length glass columns. The glass columns are packed with biochar (bed depth, 20 cm, pore volume, 14 mL) between two supporting layers of polyester fibers and glass beads.^13^ The adsorption columns were operated at room temperature and fed via a peristaltic pump (L/S Digital pump drives 07522-20 MASTERFLEX, Cole-Parmer Instrument Company) programmed at a constant volumetric flow rate. Initially, biochar inside the columns was washed with DDW; then, the AV solution was poured. Column samples were collected at different time intervals from 2 to 120 min and analyzed for dye removal. Biochar was also used to remove dyes from wastewater from silk dyeing cottage industries. The composition of the wastewater and dyeing process is given in the Supporting Information (SI).

## 3 Results and Discussion

### 3.1 Characterization of Biochar

XRD spectrum was utilized to explore the phase structures of the biochar. Figure S2 illustrates the XRD pattern of biochar with two broad peaks located at ∼24° 2θ and ∼43° 2θ corresponding to the diffraction of graphite carbon (002) and (100), respectively, and their weak intensities indicate the low degree of crystallinity.^25^ No sharp peaks were observed, demonstrating that the biochar samples are amorphous.

**Figure 1:**
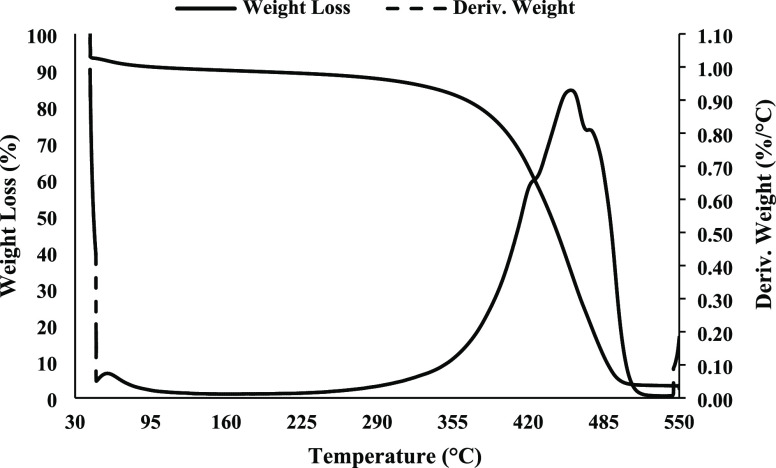
TGA and DTG of Biochar

This study’s biochar bulk density is ∼0.1 g/cm^3^, and the porosity is ∼74%, which is similar to the literature.^26^ The pH of the biochar used is basic (pH = 8.2) probably due to separating the alkali salts from organic materials during the pyrolysis.^27^ The EDS analysis (Table S2) shows that the total wt % of C in biochar is 90.94, indicating the excellent quality of the biochar.^28^ The Brunauer–Emmett–Teller (BET) surface area, pore-volume, and pore size are 306 m^2^/g, 0.146 cm^3^/g, and 1.49 nm, respectively (Table S2), which are similar to BET results of different biochar types in the literature.^29^ The weight loss versus temperature of the biochar is shown in Figure 1. The initial weight loss at 50–60 °C is probably due to moisture. After that, the weight of the biochar remains almost constant at around 90% as the temperature rises. The broad peak between 420 to 470 °C in the derivative thermogravimetric (TGA) analyses correspond to this mass loss. This mass loss is due to the degradation and decomposition of organic matter and carbon that is probably converted to CO_2_, CO, and CH_4_ and other additional substances that can appear in small amounts.^30^ A small shoulder at 420 °C can be noticed from the TGA curve, which is considered to be the decomposition of cellulose from the pinewood biomass.^31^ The prominent peak at 460 °C and the small right shoulder at 470 °C are likely attributed to lignin and lignin-like structures decomposition. The ash content will be the percentage of the biochar that remained after the decomposition of the organic fractions, i.e., the constant weight after holding the biochar at a constant temperature of 550 °C. The calculated ash content is 2.5%; this low value indicates the excellent quality of the biochar and is in line with the high carbon content of (90.94% C) shown in (Table S1).

**Figure 2:**
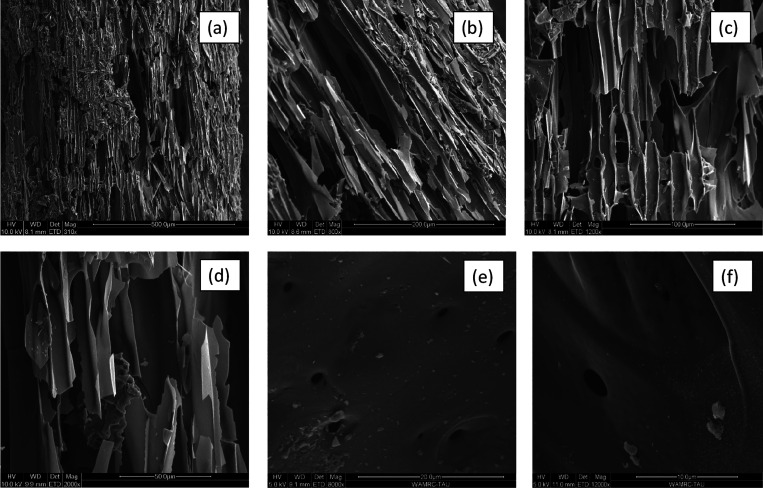
ESEM images of the biochar surface at different magnifications: (a) 310×, (b) 800×, (c) 1200×, (d) 2000×, and (e) 8000× before adsorption of AV and (f) 12,000× after adsorption of AV.

ESEM images in the biochar surface at five different magnifying levels (310, 800, 1200, 2000, and 8000×) are illustrated in Figure 2. The biochar has a microporous structure with many channels, pores, and tiny holes. This structure of the biochar is responsible for its vast surface area, which allows it to be a good absorbent material. Figure 2f shows the ESEM of biochar after absorption of AV, with no changes in the biochar morphology even after absorption.

**Figure 3:**
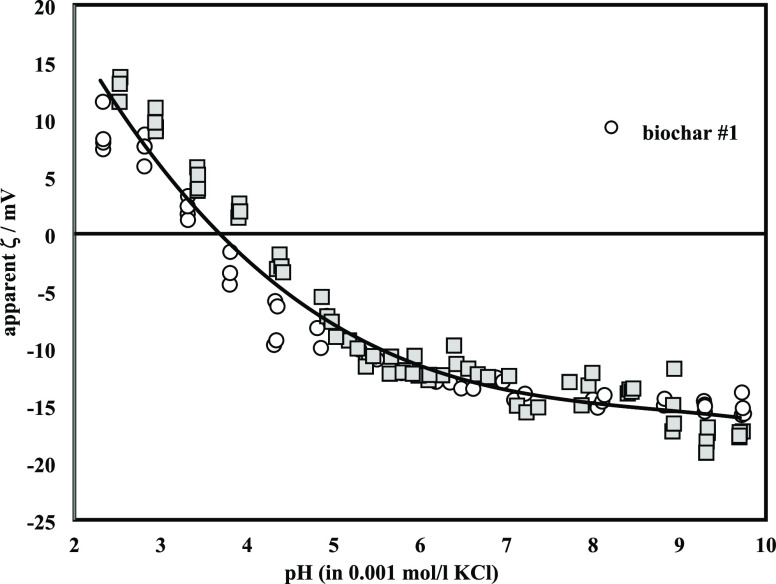
pH dependence of the apparent zeta potential for biochar.

Figure 3 shows the zeta potential for the two batches of the biochar sample in a pH range between 2 and 10. The reproducibility of the zeta potential for these two batches of biochar samples is comparable. The isoelectric point (IEP) is located at pH 3.7 ± 0.2. In the pH range above the IEP, the negative zeta potential increases toward an almost steady value of ζ = −(16.3 ± 1.4) mV at pH 9.7 in Table S1 and Figures S4 and S5.

### 3.2 Effect of pH

The effect of pH on removing AV (50 g/L) using biochar over a pH range of 3 to 9 (Figure S6) was studied. The % dye removal at pH 3, 5, 7 and 9 are 73, 65, 60, and 53, respectively. AV is an anionic dye, and due to the negatively charged SO_3_^–^ group, a positive charge on the biochar surface will result in a higher and better dye adsorption ability. At higher solution pH, the concentration of OH^–^ ions increases, competing with the dye molecules on the adsorption sites.^32^ The amount of H^+^ ions increases at acidic pH, and the electrostatic attraction between the dye and the biochar is favorable. The same trends were obtained in the literature with different acid dyes and types of absorbents.

An additional reason for a better AV removal at low pH values is the zeta potential of the biochar. Also, Figure 3 shows that the zeta potential is positive (IEP = 3.7). As the pH increases above 3.8, the number of negatively charged sites on the biochar increases, and the number of positively charged sites decreases.^3^ Because of the electrostatic repulsion between the opposing biochar surface and negatively charged dye, the surface sites on the biochar did not favor the adsorption of anionic dye. At pH 3 the highest removal efficiency occurred and was hence chosen for further experiments. After the biochar adsorbed the dye, the pH of the solution slightly changed. From initial pH 3, 5, and 7, it increased to 4.64, 7.49, and 7.56, respectively, and from initial pH 9, it decreased to 7.98. The change in the pH is due to the inherent pH of the biochar itself. From (Table S1), the natural pH of the biochar is between 8–9; after agitation of the dye solution with the biochar, the pH values tend to become closer to the natural pH of the biochar.

### 3.3 Biochar Dose Effect

**Figure 4:**
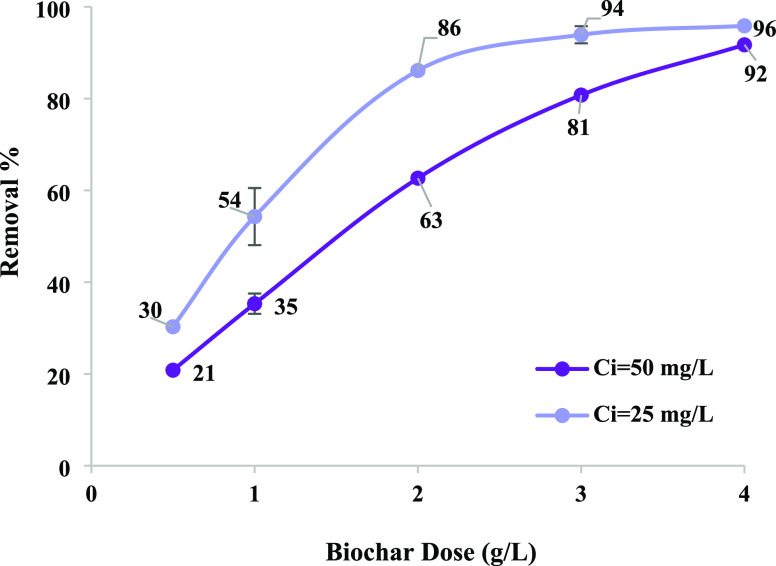
Effect of biochar dose on the removal of AV (batch, *T* = 25 °C, *t* = 240 min, pH = 3).

Figure 4 shows the removal of AV as a function of biochar dose after 4 h of agitation in batch experiments. Higher dye removal was obtained with an increase in biochar dose due to more adsorbent sites on its surface area.^33^ An adsorbent dose of 2 g/L showed optimum dye removal; consequently, this concentration was chosen for further studies. The biochar used in the current study shows superior performance compared to other studies (Table 1).

**Table 1:** Comparison of Dye Removal Studies with Biochar in Batch Reactors

dye	biochar dose	agitation time	dye uptake	removal	ref
congo red (500 mg/L)	1 g/L	24 h	51.5 mg/g	10.3%	(24)
acid red 1 (50 mg/L)	5 g/L	2 h	9.8 mg/g	97.6%	(3)
patent blue (50 mg/L)	10 g/L	1 h	3.7 mg/g	74%	(22)
reactive red 141 (500 mg/L)	1 g/L	80 min	130 mg/g	26%	(34)
acid orange 7 (20 mg/L)	3.2 g/L	10 min	6 mg/g	95.2%	(35)
methyl orange (75 mg/L)	1 g/L	2 h	38.3 mg/g	51.13%	(36)
acid red (100 mg/L)	0.4 g/L	11.6 h	115.8 mg/g	46.3%	(37)
congo red (100 mg/L)	50 g/L	84 h	2 mg/g	98.8%	(38)
acid violet 17 (50 mg/L)	2 g/L; 4 g/L	4 h	16.4 mg/g; 12 mg/g	63%; 92%	present study

### 3.4 Adsorption Kinetics

The effect of agitation time on dye removal using 2 g/L biochar at 25 and 50 mg/L of AV is shown in Figure S7. At the first 30 min, the dye adsorption rate is very high. After 30 min agitation, for dye concentrations of 25 and 50 mg/L, the dye removal was >50 and 30%, respectively. A plateau in the sorption rate was obtained after ∼360 min for an initial dye concentration of 25 and 50 mg/L. At the beginning of the adsorption process, the number of empty adsorption sites is numerous, which results in a faster dye adsorption rate. After the first 30 min, the adsorption sites on the biochar surface become more saturated. Thus, dye removal rate becomes slower. The initial dye concentration is an important parameter that influences adsorption. The concentration gradient between the solution and adsorbent is higher at higher dye concentration, which is the driving force to carry dye molecules from the dye solution to the dye solution’s biochar surface. This can also explain the faster and greater adsorption at the initial period of the process.^39^

**Figure 5:**
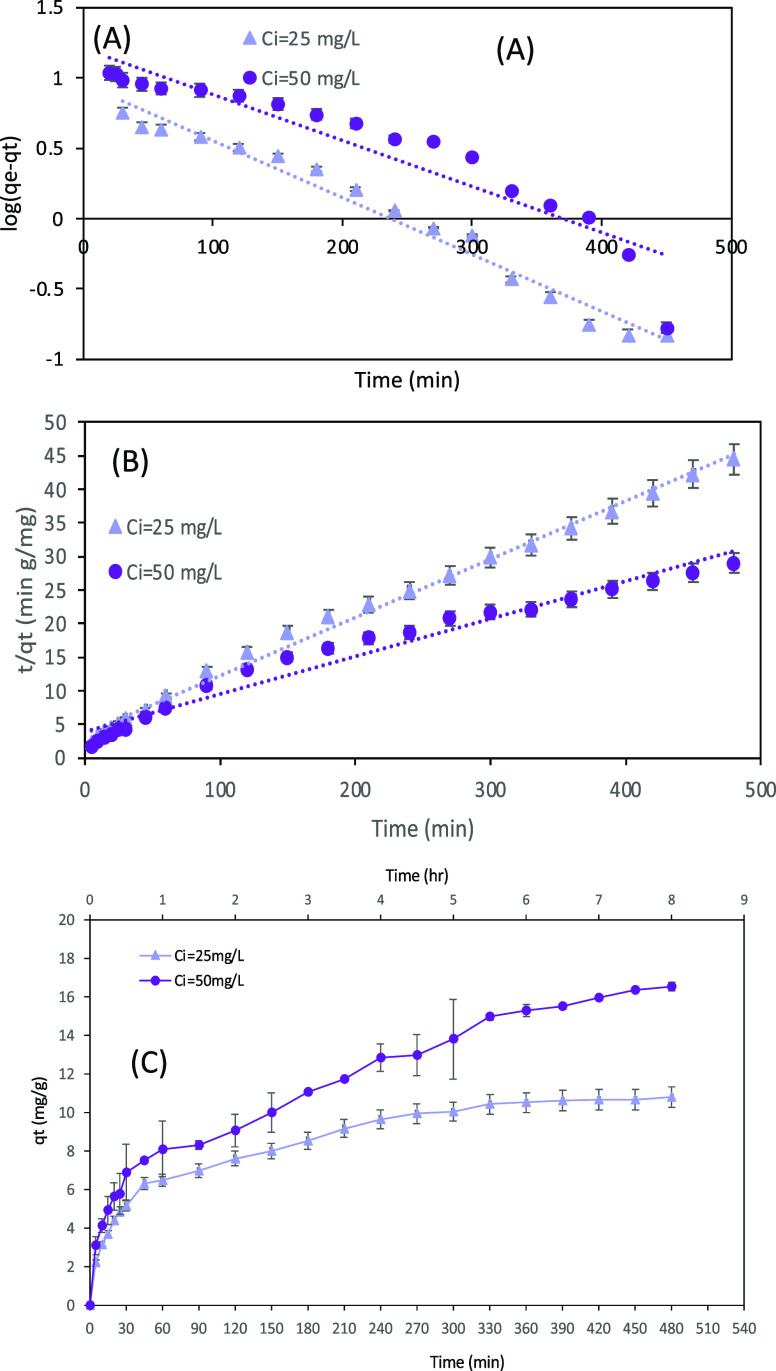
The kinetic models of the obtained batch experiments for AV removal with biochar under conditions of *T* = 25 °C, pH = 3, and *m* = 2 g/L. (A) Pseudo-first-order model. (B) Pseudo-second-order model. (C) Intraparticle diffusion (IPD) model.

Three kinetic models were examined using kinetic adsorption experiments to understand the adsorption mechanism and dynamics. The data from Figure S7 was implemented in eq 4 for the pseudo-first-order model, eq 5 for the pseudo-second-order model, and eq 7 for the intra-particle diffusion (IPD) model. All three models were plotted in Figure 5, and all the parameters from these equations are summarized in (Table 2). However, Figure 5,BA shows the plots for pseudo-first- and pseudo-second-order models for AV adsorption onto the biochar. According to the *R*^2^ parameter (Table 2), the best fit model is the pseudo-second-order for both initial concentrations 25 and 50 mg/L. From the literature, at low initial concentrations (tens of mg/L) as examined in this research, the most fitted model is the pseudo-second-order. In contrast, for high initial concentrations (hundreds of mg/L), the pseudo-first-order model usually fits better.^19^ The calculated dye uptake value, *q*_e_, for *C*_i_ = 25 mg/L was closer to the obtained experimental *q*_e_ value (10.80 mg/g) for the pseudo-second-order. In contrast, for *C*_i_ = 50, mg/L the calculated *q*_e_ by the first-order model was closer to the experimental one (16.52 mg/L). As the governing kinetic model is the pseudo-second-order, the adoption process is based mainly on chemical interactions between the biochar surface and dye ions.

**Table 2:** Kinetic Parameters for AV Dye Removal with Biochar (*T* = 25 °C, pH = 3, *m* = 2 g/L)

kinetic model	parameter		
pseudo-first-order	*C*_i_ (mg/L)	25	50
	*q*_e_(exp) (mg/g)	10.80	16.52
	*q*_e_(cal) (mg/g)	9.01	16.29
	Δ*q* (%)	16.57	1.39
	*K*_1_ (min^–1^)	0.0092	0.0076
	*R*^2^	0.9738	0.8847
pseudo-second-order	*C*_i_ (mg/L)	25	50
	*q*_e_ (exp) (mg/g)	10.80	16.52
	*q*_e_(cal) (mg/g)	11.55	17.73
	Δ*q* (%)	6.94	7.32
	*K*_2_ (g/mg min)	0.0021	0.0121
	*h* (mg/g min)	0.2764	0.2627
	*R*^2^	0.9942	0.9668
intraparticle diffusion	*C*_i_ (mg/L)	25	50
first stage	*K*_p_ (mg/g min^–0.5^)	0.9127	1.1385
	θ (mg/g)	0.2252	0.5446
	*R*^2^	0.9974	0.9997
second stage	*K*_p_ (mg/g min^–0.5^)	0.3813	0.6364
	θ (mg/g)	3.5322	2.7812
	*R*^2^	0.9893	0.9820
third stage	*K*_p_ (mg/g min^–0.5^)	0.0875	0.4390
	θ (mg/g)	8.8508	6.9476
	*R*^2^	0.9228	0.9875

The pseudo-second-order model assumes that the rate-limiting step is chemisorption, which includes chemical and valence forces by exchanging electrons between the adsorbent and the adsorbate. In contrast, the pseudo-first-order model indicates physical sorption. Figure 5C shows the plot for the IPD model. The plots are not linear and can be separated into three linear zones that indicate multiple stages of adsorption. From Table 2, the *R*^2^ values of the linear regions are above 0.9, implying that the IPD model can characterize adsorption. These linear lines do not pass through the origin. Hence, the IPD is not the only rate-limiting factor.

The intercept θ in eq 7 and the plot of Figure 5C represent the thickness of the boundary layer. When θ is zero, there is no boundary layer and the IPD is the sole rate-controlling step, but if it is not zero, as in this study, more mechanisms besides the IPD can affect adsorption. θ expresses the thickness of the boundary layer and can resemble the viscous boundary layer in fluid mechanics, which is not negligible with high drag force. Around the biochar surface, there is a thin film of the bulk solution surrounding it. The dye molecule will have to diffuse through this zone to reach and approach the pores of the biochar. This slows the dye adsorption and makes it more difficult.

In the first stage of the adsorption, the θ value is small. However, at the second and the third stage, θ becomes bigger, i.e., the boundary layer got more prominent in correlation with the lower dye removal after the first 30 min of adsorption. Furthermore, at the first stage, θ is lower for *C*_i_ of 25 mg/L. However, later, the opposite occurs, and θ is lower for *C*_i_ of 50 mg/L. The concentration gradient pressure is probably the driving force that carries dye molecules to the biochar surface. When the initial concentration of the dye is higher, this driving force for adsorption is higher.^21^

### 3.5 Effect of Temperature and Concentration of Dye

**Figure 6:**
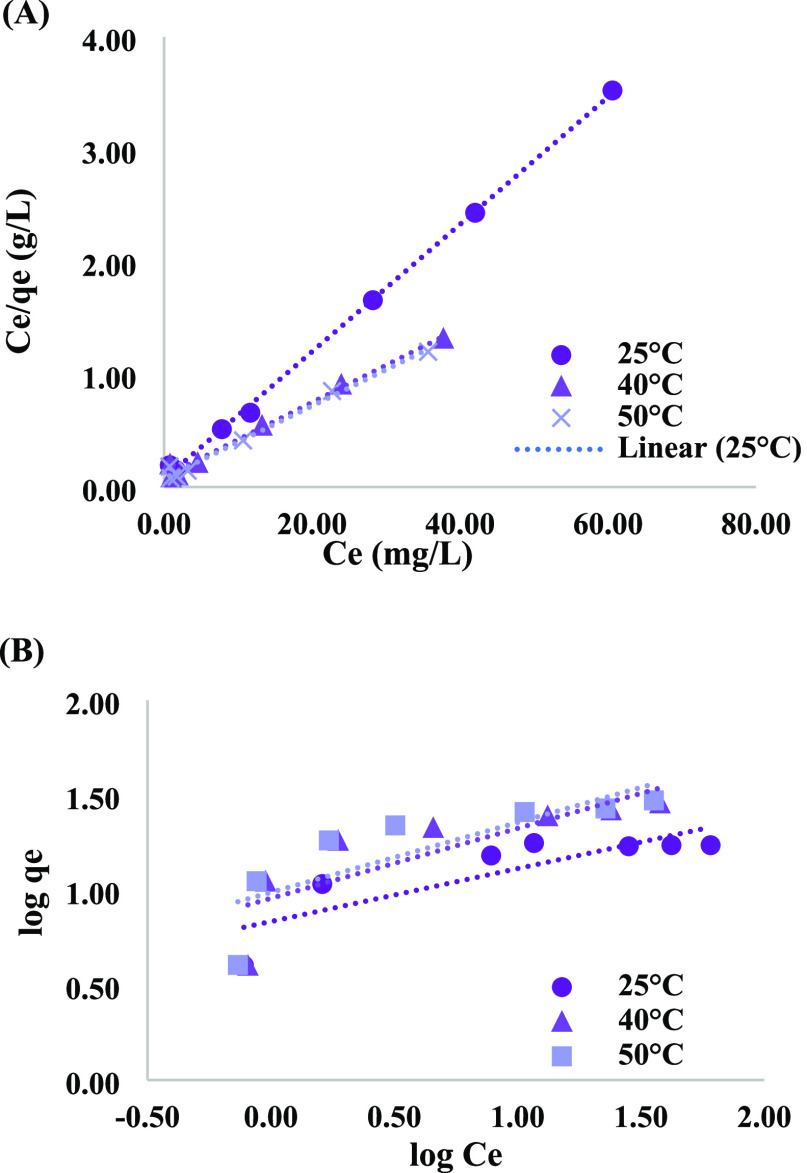
(A) Langmuir and (B) Freundlich adsorption isotherm of AV onto biochar (*t* = 480 min, pH = 3, *m* = 2 g/L). However, after 40 °C, the increase in the rate of absorption is negligible. The same trend was obtained for the other acid dyes in the literature. The obtained models’ constants and correlation coefficient *R*^2^, summarized in Table 3, show that the Langmuir isotherm is fitted considerably better than the Freundlich isotherm. Langmuir adsorption isotherm indicates that the dye molecules are adsorbed on the biochar surface in a monolayer and homogeneous structure.

Figure 6A,B shows the Langmuir and Freundlich adsorption isotherms at three different temperatures. For higher operating temperatures, the adsorption is better. The maximum dye uptake almost doubled with the increase in temperature from 25 to 40 °C, indicating an endothermic sorption process in nature.^41^

The monolayer sorption mechanism characterizes the chemisorption processes, which is correlated to increased AV removal with increased temperature. *R*_L_, the Langmuir separation factor, is <1 for all the examined temperatures, pointing to a favorable adsorption isotherm of AV onto biochar surface.^42^ In Table 3, the calculated maximum adsorption from the Langmuir isotherm, *q*_m_, was well correlated to the experimental values since the relative error Δ*q* was very small and insignificant. These emphasize the excellent compatibility of the Langmuir model to the AV adsorption to the biochar.^43^ Responsive surface methodology (RSM) was adapted to optimize the experimental parameters for achieving maximum dye removal by the biochar. In the RSM approach, batch runs were conducted in CCD model-designed experiments to visualize the effects of independent factors on the response and the results along with the experimental conditions. This is explained in detail in the Supporting Information. The developed model suggests that initial dye concentration of 25 mg/L, pH 3, 2 g/L biochar, and sorption time of 240 min are optimum conditions for maximum color removal of biochar.

**Table 3:** Isotherm Parameters for Langmuir and Freundlich Models for AV Removal with Biochar

isotherm model	parameter			
Langmuir	*T* (°C)	25	40	50
	*K*_L_ (L/mg)	0.79	0.37	0.42
	*q*_m_ (mg/g)	17.67	30.21	31.06
	*q*_exp_ (mg/g)	17.71	28.67	29.70
	Δ*q* (%)	0.23	5.37	4.58
	*R*_L_	0.01	0.03	0.02
	*R*^2^	0.9988	0.9902	0.9891
Freundlich	*T* (°C)	25	40	50
	*K*_F_ (mg/g)(L/mg)^1/*n*^	0.16	0.64	0.89
	1/*n*	0.28	0.37	0.37
	*R*^2^	0.7166	0.6759	0.6599

### 3.6 Adsorption Thermodynamics

The thermodynamic plot for the adsorption of AV dye onto the biochar for initial concentrations of 25 and 50 mg/L at three operating temperatures (25, 40, and 50 °C, corresponding to 298, 313, and 323 K, respectively) is shown in Figure S8. The thermodynamics parameters were calculated from these graphs using eqs 13, 14, and 15 and are listed in Table 4.

**Table 4:** Thermodynamic Parameters of AV Adsorption onto Biochar

thermodynamic parameters	temperature (K)	25 mg/L	50 mg/L
Δ*G*° (KJ/mol)	298 (25 °C)	–4.70	–1.03
	313 (40 °C)	–6.39	–4.01
	323 (50 °C)	–6.83	–5.17
Δ*H*° (KJ/mol)		21.48	49.35
Δ*S*° (KJ/mol K)		0.088	0.169

The change in the free energy, Δ*G*°, is negative, indicating that AV’s adsorption process on the biochar is spontaneous and favorable. However, the absolute value of Δ*G*° demonstrates increased temperature due to the increased affinity of AV to the biochar at higher temperatures.^40^ The positive values of the entropy change Δ*S*°, indicating the increase of randomness and disorder at the solid–liquid interface during the adsorption and the positive values of the enthalpy change Δ*H*° pointing again on an endothermic process with a correlation of better dye adsorption at higher temperatures as mentioned earlier.

### 3.7 Adsorption of Ternary Dye Mixtures by Biochar

The biochar’s selectivity was determined by testing the adsorption of dyes on the biochar using a mixture of AV, acid green (AG), and methylene blue (MB). The trend in adsorption showed that MB (92) > AV(86) and AG (70) (Table S6). MB is a cationic dye that possesses a positive charge in an aqueous medium, whereas AV and AG are anionic dyes. The pH of the biochar is in the range of 8–9 and has a negative charge as explained in Section 3.2, thus favoring MB due to its positive charge. These experiments imply that electrostatic interactions are the driving force in the adsorption mechanism of biochar.

### 3.8 Regeneration

AV adsorption onto regenerated biochar is shown in Figure S9. Biochar regeneration was done by sonication of used biochar with absolute ethanol for five cycles. After each regeneration, a batch adsorption experiment was conducted to estimate the biochar regeneration ability with 50 mg/L AV solution and a biochar dose of 2 g/L at 25 °C. The highest adsorption rate occurred during the first 30 min of agitation; hence, the adsorption experiment after the regeneration was tested for 30 min. As seen in Figure S9, at the initial adsorption (nonused biochar), after 30 min, the removal is ∼30%. The adsorption after the regeneration studies was very close to the initial 30% for all the five cycles, reducing only 3% from the initial dye removal after the fifth cycle as shown in Figure S11 (relative error of 10.3% between the initial and the lowest adsorption in the fifth cycle). Figure S12 shows that the biochar can be reused for at least 5 cycles without losing its absorption capacity.^17^ The initial mass used was 0.101 g biochar; after five cycles of regeneration, the mass of biochar after regeneration was 0.098 ± 0.001 g, with a relative error of 3.5% compared to the initial mass. Although the mass loss is almost negligible, it may decrease the dye removal after several regenerations.

### 3.9 Fixed-Bed Column Study

**Figure 7:**
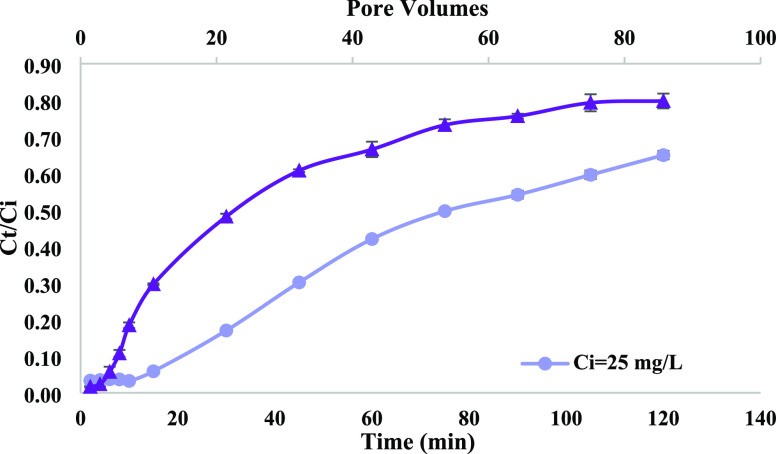
AV adsorption experiment with the column studies.

AV removal under continuous flow studies was performed using sorption columns with dye concentrations of 25 and 50 mg/L and a constant flow rate of 10 mL/min for 120 min.^32^Figure 7 presents *C_t_*/*C*_i_ as a function of filtration time or as a function of pore volumes. After 120 min and 86 pore volumes, the concentration of the effluent was around 80% of the initial 50 mg/L (*C*_120min_ = 40 mg/L) AV and about 65% of the initial 25 mg/L dye concentration (*C*_120min_ = 16 mg/L). As expected, the concentrations of the influent and the effluent is lower than the experimental condition. It takes time to reach saturation of the biochar adsorption sites when the influent concentration is lower due to less competition for the dye to reach the biochar surface. After 120 min, the dye uptake for the initial 50 mg/L was higher than the uptake of the 25 mg/L (*q*_50mg/L_ = 14.64 ± 0.45 mg/g, *q*_25mg/L_ = 11.36 ± 0.14 mg/g). An increase in adsorption capacity can be due to the higher driving force for mass transfer at higher dye initial concentration. The adsorption capacity of various dyes on different adsorbents reported earlier is presented in Table 5. In all cases, the influent flow rate is lower in the present study; this can be one of the reasons for lower dye uptake and the smaller absorbent quantity used in this study.

**Table 5:** Adsorption Capacity of Various Adsorbents for Dye Removal in a Column System

dye	adsorbent amount	flow rate (mL/min)	max dye uptake (mg/g)	reference
acid violet 17 (200 mg/L)	6 g of biosorbent from *Ficus racemosa* leaves	8	69	(45)
malachite green (88 mg/L)	0.1 g of fibrous cellulose sulfate from medical cotton waste	5.6	815	(46)
methylene blue (40 mg/L)	112 g of sugarcane biochar entrapped in calcium alginate	2.5	30	(47)
acid violet 17 (50 mg/L)	1.5 g of pine tree biochar	10	15	present study

**Table 6:** pH of Different Effluent Samples before and after Treatment with GAC 1240 and Biochar

		after treatment	
effluent sample	before treatment	GAC	biochar
dye	9.12	9.67	9.69
acid wash	4.60	4.74	4.90
wash water	7.71	9.11	9.08
mixed	9.15	9.43	9.52

### 3.10 Batch Experiments with Real Textile Wastewater Effluent

Batch experiments were conducted with all three types (acid/wash and dye effluents) of the silk dyeing process, separately, and with the mixture of all three of them together (Figure S13). The experiments were conducted at ambient temperature with the loading of 1 g biochar on 100 mL effluent. As was expected, all the pH values of the effluents are quite basic except for the acid wash that contains acetic acid. After the treatment, the pH did not change significantly; most of the values increased a bit, apparently because of the fundamental nature of the biochar (Table S2) and the granular activated carbon (GAC Norit 1240).^44^ The wash water effluent pH increased the most, from pH 7.7 to around pH 9.

The dye removal percentage was determined proportionally by comparing the absorbance of the spectroscopic measurements in the visible spectrum of the light before and after the treatment (Figure S11). The comparison was made with biochar and GAC Norit after 30 and 60 min of sorption treatment. The adsorption with the biochar examined was much more efficient compared to GAC (Figure S14). However, comparing the different effluents, the highest removal is observed for acid and wash water. The pH plays a role in the dye removal, as observed in Figure S6, as lower pH increases adsorption. The pH of the acid wash is the lowest of all the effluents; around pH 4, the dye removal is the best, and the pH of the wash water is around 7. Thus, the removal is good but not as good as the removal of the acid wash. The pH of the dye and the mixed water samples are ∼9, which explains the lowest removal for these effluents.

The color of the dye and the mixed water samples was very dark, indicating that these samples are very concentrated, which can result in lower removal. Another reason for the low removal in the dye water is the high presence of the sericin released from the silk filament to the boiling water of the dye bath effluent. The sericin makes the water more viscous and interferes with the adsorption process.^48^ The viscosity of the sericin probably expands the boundary layer. This viscous layer surrounding the biochar surface area makes it harder for the dye molecules to approach it and be adsorbed. The same phenomenon can be the reason for poor dye removal in the mixed water effluent, since the mixed water also contains viscous dye water with the sericin.

### 3.11 Suggestion for a Textile Wastewater Treatment System Design

**Figure 8:**
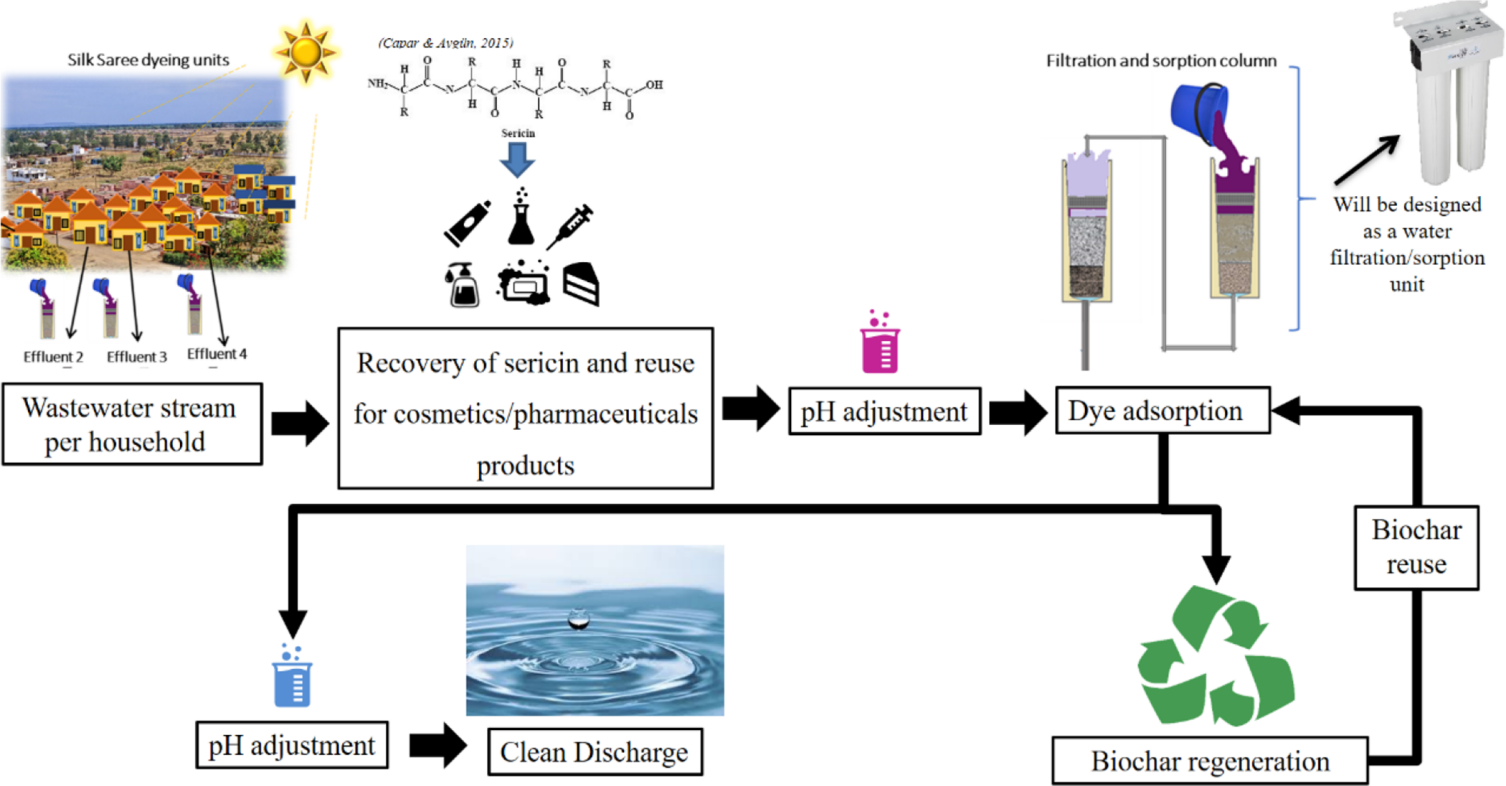
Process flowchart for the textile dye effluent adsorption system.

A process flowchart for the textile dye effluent adsorption system is demonstrated in Figure 8 and Figure S15 according to the results from the artificial dye solution and actual textile effluents. Due to the high total suspended solids (TSS) values, microfiltration will be the first step required to reduce TSS load. The sericin released from the silk filament interferes with the dye adsorption process and leads to high TSS and exerts a high oxygen demand in the effluent. Thus, before the dye adsorption, the sericin needs to be removed and can even be reused.

The protein sericin became very popular recently due to its biological properties, and it is applied in the food industry, medical and pharmaceutical fields, and cosmetic products. Different processes can recover sericin from silk wastewater, such as ultrafiltration, precipitation, enzymatic hydrolysis, freezing, and tray drying. Sericin recovery from textile wastewater effluent can be one of the steps in the dye removal process, thereby producing another product and the sarees.

For efficient dye removal, pH reduction is needed. Dye adsorption via biochar can be done in columns with a continuous flow as commonly used in many wastewater plants or as a batch reactor treatment system with an agitation tank with a mixer. After the biochar adsorbed the dye, a regeneration of the biochar with ethanol will be done to reuse the biochar several times and make the process more environmental. The clean water after the biochar treatment can be used again in the dyer houses for the dyeing process instead of discharging it to the environment.

## 4 Conclusions

The current study exhibits the suitability and conditions of using pine tree biochar for the adsorption of acid dyes from real textile wastewater effluent and the adsorption of AV from the synthetic dye. After 30 min of agitation with 2 g/L biochar in dye concentrations of 50 and 25 mg/L at room temperature, 30–40% of the AV dye was removed. In contrast, almost all the dye was removed from real textile effluent with a biochar dose of 10 g/L. Kinetic studies showed that the pseudo-second-order model is the one that best describes the adsorption process. A Langmuir isotherm fits the sorption of AV to biochar with maximal dye uptake of 29 mg/g at 40 °C. Five cycles for reusability of the biochar for AV dye removal were demonstrated. Biochar was successfully adapted to remove the color from real textile effluents from silk dyeing units.

## Supporting Information Available

Additional experimental details and figures, including biochar characterization; FT-IR spectra; PXRD patterns; physicochemical properties of biochar in the presence of AV dye; thermodynamic plot (PDF)

## Notes

E-supplementary data of this word can be found in the online version of the paper.
