# Supplementary material for: Effective Removal of Acid Dye in Synthetic and Silk Dyeing Effluent: Isotherm and Kinetic Studies
Source: ACS Omega. 2021 Dec 30;7(1):118–28. doi: 10.1021/acsomega.1c04111 (PMC8757339; doi:10.1021/acsomega.1c04111)
Supplement: Supplementary file 1 — ao1c04111_si_001.pdf [file ao1c04111_si_001.pdf]

Supporting Information

Effective removal of acid dye in synthetic and silk dyeing effluent: isotherm and kinetic studies

Elizaveta Sterenzon[a], Vinod Kumar Vadivel[a*], Yoram Gerchman[b], Thomas Luxbacher[c,d], Ramsundram Narayanan[e] and Hadas Mamane[a]

[a]School of Mechanical Engineering, Faculty of Engineering, Tel Aviv University, Tel Aviv 69978, Israel

[b]Department of Biology and Environment, Faculty of Natural Science, University of Haifa and Oranim College, Tivon 3600600, Israel

[c]Anton Paar GmbH, Anton-Paar-Str. 20, 8054 Graz, Austria

[d]Faculty of Chemistry and Chemical Engineering, University of Maribor, 2000 Maribor, Slovenia

[e]Department of Civil Engineering, Kumaraguru College of Technology, Coimbatore, Tamilnadu 641049, India.

Corresponding Author Email: *vinodkumarv@mail.tau.ac.il*

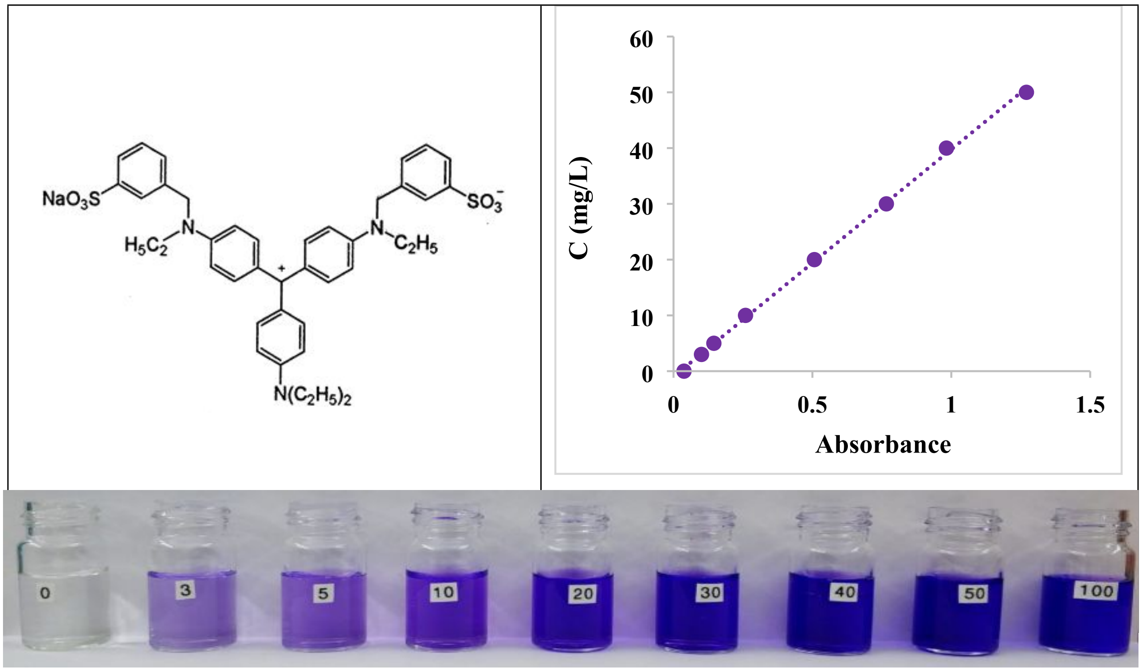

Figure S1: Calibration curve for AV and Molecular structure of Acid Violet 17 (AV).

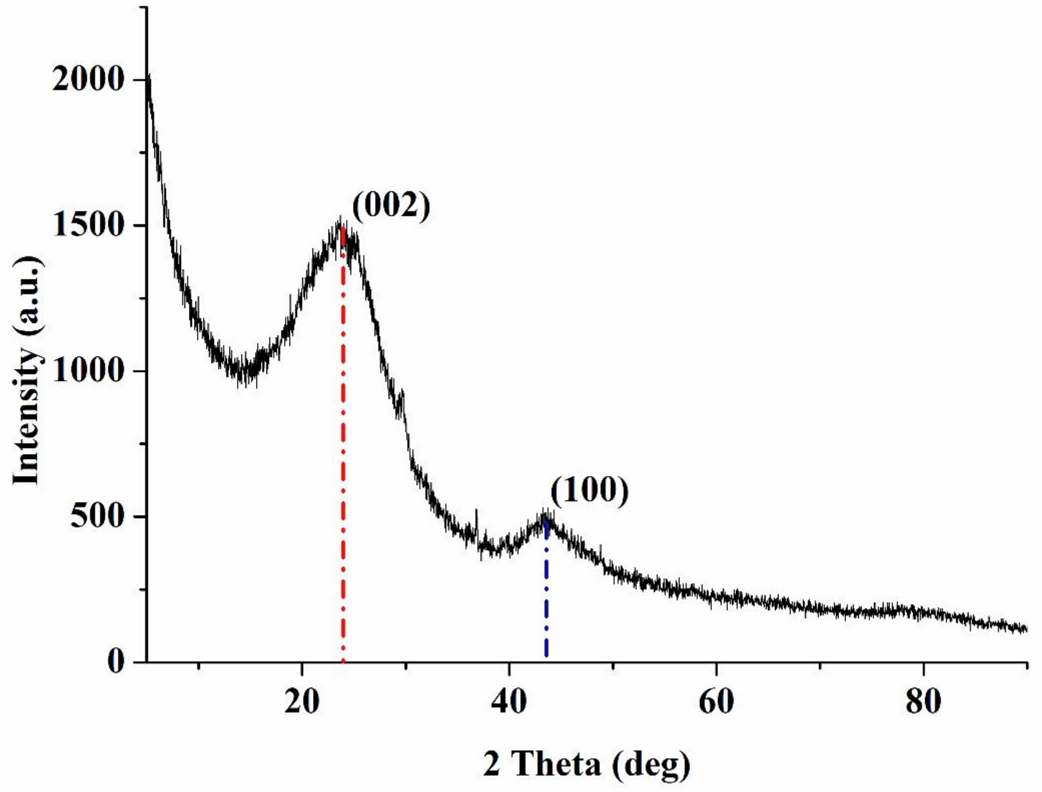

Figure S2: XRD pattern of pine-biochar.

1. Dyeing process of soft silk and Kovai Kora Sarees

For the dying of silk filaments, acid dyes solutions are used in big vessels. The acid dyes contain sodium salt from sulfonic acid what makes them highly soluble in water[1]. In a mixture of water and acetic acid [2], the NH_2 groups of the silk fibers are protonated to give a positive charge of NH_3^+, allowing the formation of ionic interactions between the fibers the negative dye charge. Van-der-Waals bonds, dipolar bonds, and hydrogen bonds are also formed between the dye and the fiber[3]. For the dyeing, acid dye powder is dissolved in water, followed by soap and washing soda to remove the sericin [4] better. Sericin is a protein present on the surface of the filament of the silk and is responsible for its stiffness/ harshness. Thus, removing it makes the silk softer[5].

Then the dye bath temperature is raised to boil. At boiling, the fiber dyeing is continued for 5 to 10 minutes until the required shade is attained. However, to fix the dye on the surface of the silk fiber, the dyed silk skein is immersed in an acid bath with water: acetic acid ratio of 2:1. In acidic solution, fixation of the dye molecules onto the silk by ionic bonds is enhanced due to the electrostatic attractions [6]. After the dye fixation with an acetic acid solution, the fibers are washed with plain water to remove all the dye and acid excess and hung to dry [7]; then, the dried material will be transferred to weaving on the handloom. One of the house dyers' main challenges is the lack of a proper textile wastewater effluent collection and treatment system. The used dye solution is discharged to the drainage or environment to groundwater or the river and may cause severe environmental damage and health problems.

2. Real Effluent Characterization

Batch experiments were conducted with actual effluent samples from house dyers. The dye effluent can be divided into three categories:

1. Dyeing effluent: Water remains after the dyeing process and contains dissolved acid dye powder, soap, washing soda, and sericin from the silk fibers that make it a bit viscous.
2. Acid wash effluent: Water that remains after the fixation process. It contains acetic acid, a small amount of acid dye that is released from the dyed silk and sericin.
3. Wash water effluent: After the acid wash, the silk was washed with plain water. Hence everything that was in the acid wash can also be in this effluent.

Table S1 shows the real dye effluent properties consist of TDS and TSS (25,660 and 22,460 mg/L), respectively. However, increased TDS is due to high dye molecules and inorganic salts, and high TSS is probably due to sericin protein from the silk and small fibers that are released into the water during the dyeing. The UV absorbance at 254 nm is the highest (0.56 OD) for dyeing effluent because of the high presence of dye molecules which are organic molecules.

Also, the absorbance at 280 nm is the highest for this effluent, indicating a high sericin protein [8]. The dyeing process is done at water boiling temperature in soap and sodium carbonate (alkaline hot water). These conditions cause a degumming process, which means the sericin is separated from the silk fibers and dissolves in the water; the released sericin can also contribute to high COD in the dyeing effluent.

Table S1: Characterization of wash water, acid wash, and dyeing effluents.

Parameter	Wash water effluent	Acid wash effluent	Dyeing effluent
**L/day/household AVG**	300 – 400	~75	70 -100
**pH**	6.93	4.33	9.25
**COD (mg/L)**	2,560	16,640	18,560
**TDS (mg/L)**	17,822	21,909	25,660
**TSS (mg/L)**	9,865	16,320	22,460
**UV Abs @ 254 nm**	0.05	0.02	0.56
**UV Abs @ 280 nm**	0.04	0.02	0.50

Table S1 also presents the amount of effluent generated on an average per day from each household. The exact amount will vary depending on the quantity of dyed material per day, but approximately 500 to 600 L will be discharged to the environment /day/ household (personal communication). In the sirumugai, 80 to 100 households practice dyeing, resulting in 40,000 to 60,000 L of contaminated effluent discharged to rivers and lakes daily (personal communication). Figure S shows the dyeing process, the different dye effluents, and the discharge to the environment.

Table S2: Pine biochar properties.

Bulk density (g/cm^3)	Porosity (%)	pH	Ash content (%)	EDS analysis (wt%)				BET Surface Area (m^2/g)	BJH Pore Volume (cm^3/g)	BJH Pore Radius (nm)
				C	O	Al	K			
0.097	74.15	8.2	2.5	90.94	7.58	0.45	1.035	306	0.146	1.49

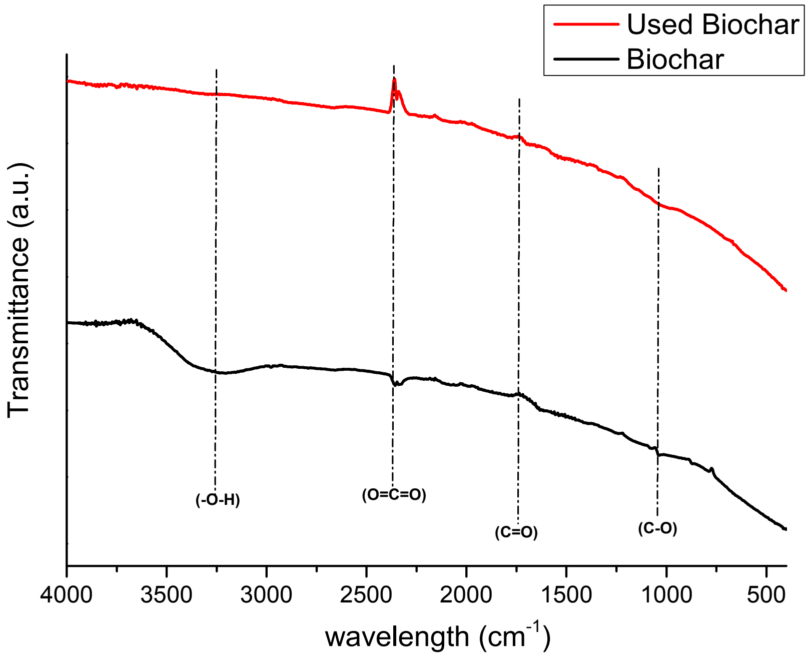

Figure S3: FTIR spectrum of non-used and used biochar after AV dye adsorption.

The elaborate explanation for Figure S4: Zeta potential results of biochar performed by the streaming potential (SP) are yet to be explored. However, we compare our data with the zeta potential determined by the electrophoretic mobility measurements (electrophoretic light scattering, ELS) of different biochars available in the literature. For this purpose, the biochar sample needs to crush and the fine particles dispersed in an aqueous solution. Figure S.4 shows the results for a series of biochar samples obtained from different straws (peanut, soybean, canola) determined in 0.0002 M NaNO3 [9.]

The zeta potential determined by ELS is significantly negative. The lower ionic strength of the background solution (0.2 mM for ELS instead of 1 mM for SP) and the different types of biochar samples contribute to this difference but are certainly not the only reasons for the discrepancy. Despite the improved evaluation of the non-linear pressure ramps discussed below, the biochar sample's conductance introduces a calculation error when applying the Helmholtz-Smoluchowski equation.

$$\zeta = \frac{dU_{str}}{\Delta p} \times \frac{\eta}{\varepsilon_{rel} \times \varepsilon_0} \times \kappa_B \quad (1)$$

The electric conductivity of the bulk aqueous solution, κB, is insufficient to represent the conductivity within the porous plug of the biochar sample. Material conductance, κe, but also the ionic conductance within the porous biochar particles, κp [10], contribute to the overall conductance such that κB in Eq. 1 needs to be replaced by

$$\kappa_{total} = \kappa_B + \kappa_e + \kappa_p \quad (2)$$

Unfortunately, the contributions κe and κp remain unknown.

(Figure S4) also shows a significant difference between the isoelectric points of straw-based biochar and our biochar sample derived from a pine tree. Again the different types of biochar samples may account for the discrepancy in the IEP. Furthermore, grinding the biochar sample created a new surface area, which is likely more reactive than granular biochar's aged surface. The lower IEP and thus the higher acidity of straw-based biochar would account for the enhanced reactivity. Note that the surface area increases enormously due to the small particle size required for ELS (≤ 1 µm). We have also ground a sample of the pine tree-based biochar to prepare a dispersion in deionized water for a comparative ELS measurement (Litesizer 500). As shown in Figure S.4 the result compares well with the zeta potential of straw-based biochar and confirms the streaming potential method's restrictions.

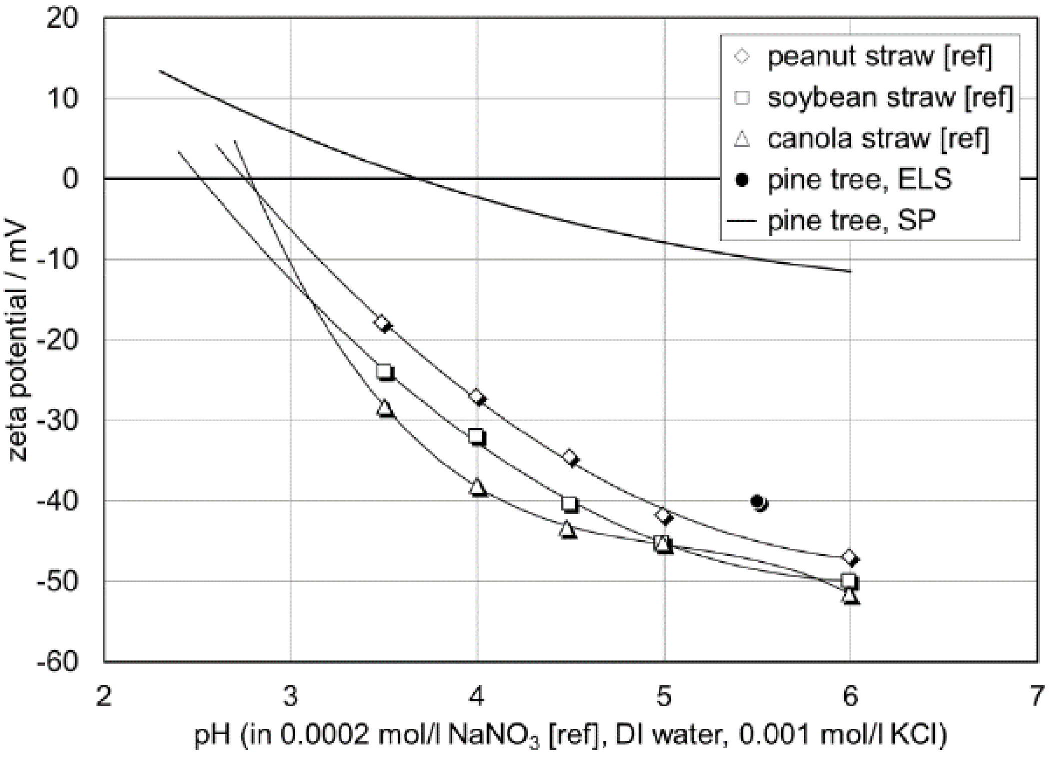

Figure S4: Comparison of apparent zeta potential for biochar derived from pine tree based on streaming potential measurement (SP) with the zeta potential for straw-based biochar determined by electrophoretic mobility measurement (ELS).

4. Analysis of non-linear pressure ramps

Figure S. 5 shows a typical example of a pressure ramp obtained for the biochar sample. The dependence of the potential streaming user on the pressure gradient Δp is non-linear. According to the classical Helmholtz-Smoluchowski approach, a linear fit gives a zeta potential of $\zeta = -8.84$ mV with an unsatisfactory linear regression coefficient of $R^2 = 0.9881$. Although the physical background of non-linear pressure ramps for material surfaces that exhibit metal (electron) conductance is not yet fully understood, the measurement of both streaming potential and streaming current (not shown) exhibits a reproducible parabolic shape of the pressure ramps. This behavior lets us conclude the polarizing effect of the streaming potential arising by the liquid flow through the (biochar) sample capillaries on the conductive (biochar) sample.

The effect is higher at high streaming potential, which occurs at higher pressure difference, and decreases as the streaming potential reduces. Ideally, the streaming potential coupling coefficient dUstr/dΔp should determine at the no-flow condition. However, this is impossible since the streaming potential is inhibited in the absence of liquid flow.

The non-linear dependence of the streaming potential on the pressure gradient represented by a parabolic polynomial fit (power 2 or power 4 depending on the pressure range considered for the analysis of the pressure ramp) and determined the streaming potential coupling coefficient as the slope of this parabolic curve at the extrapolated Δp = 0 mbar. In the example shown in Figure S.3, this approach gives a zeta potential of ζ = –16.6 mV, which is 88% higher than the zeta potential obtained from the erroneous linear fit of the measured data.

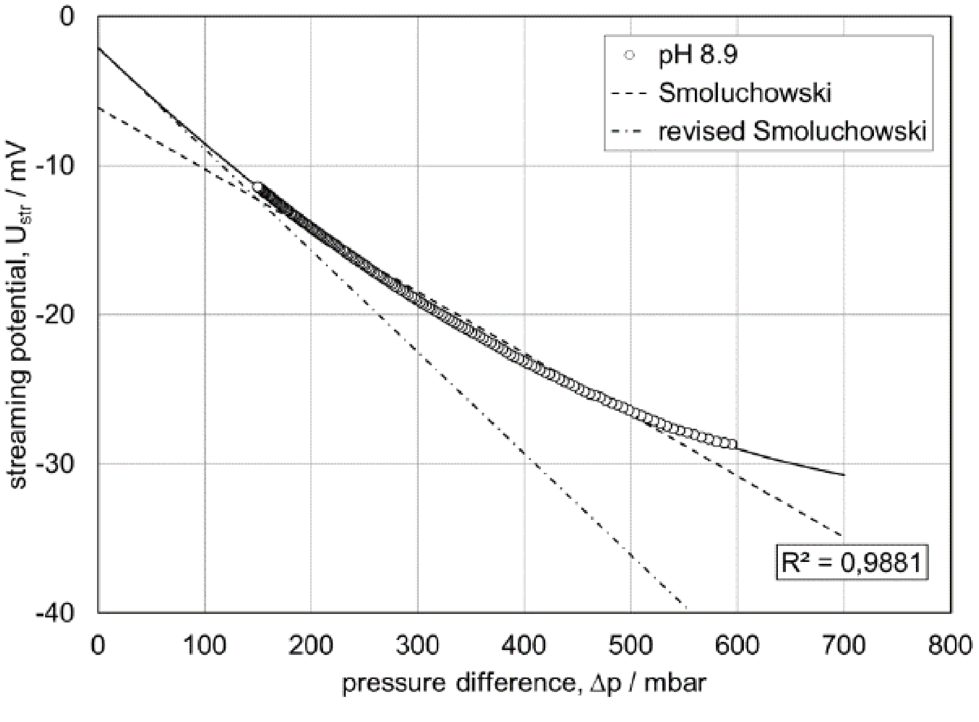

Figure S5: A typical example of a pressure ramp obtained for the biochar sample.

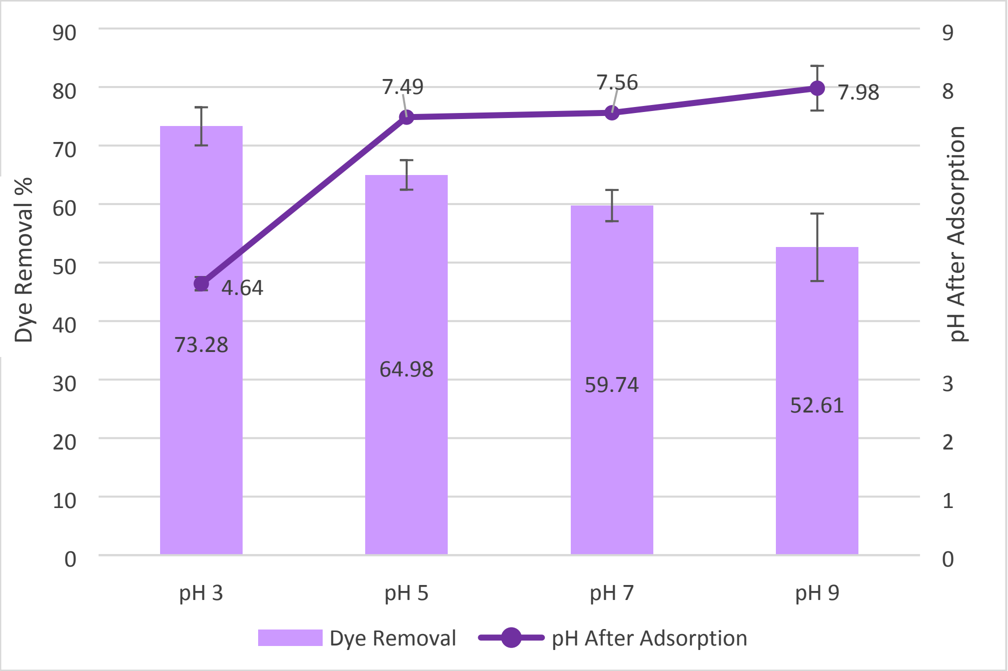

Figure S6: Effect of the initial dye solution pH on the AV's adsorption on the biochar.

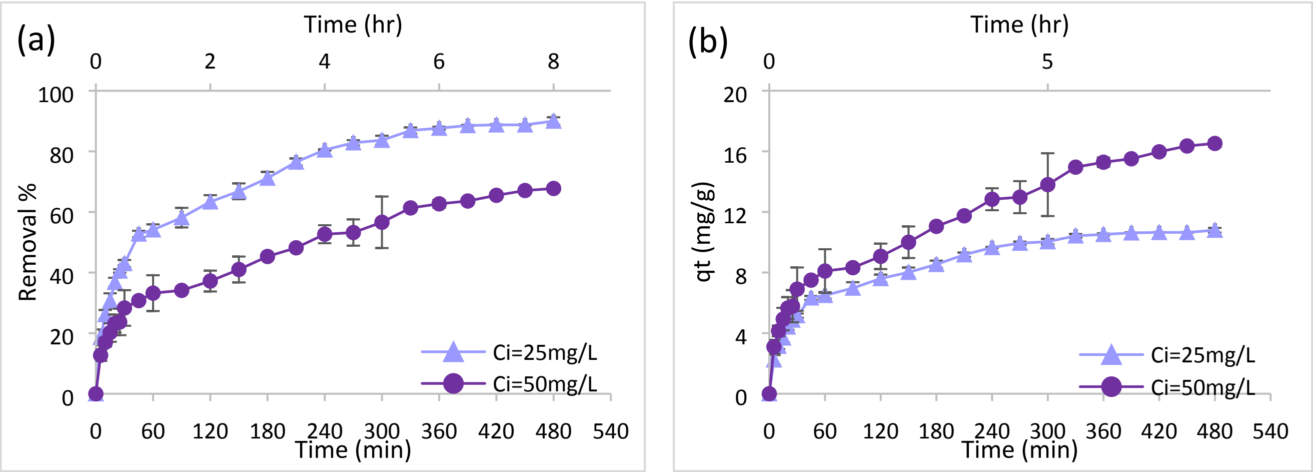

Figure S7: Effect of agitation time and initial AV concentration on the (a) dye removal and (b) dye uptake onto the biochar (T=25°C, pH=3, m=2 g/L).

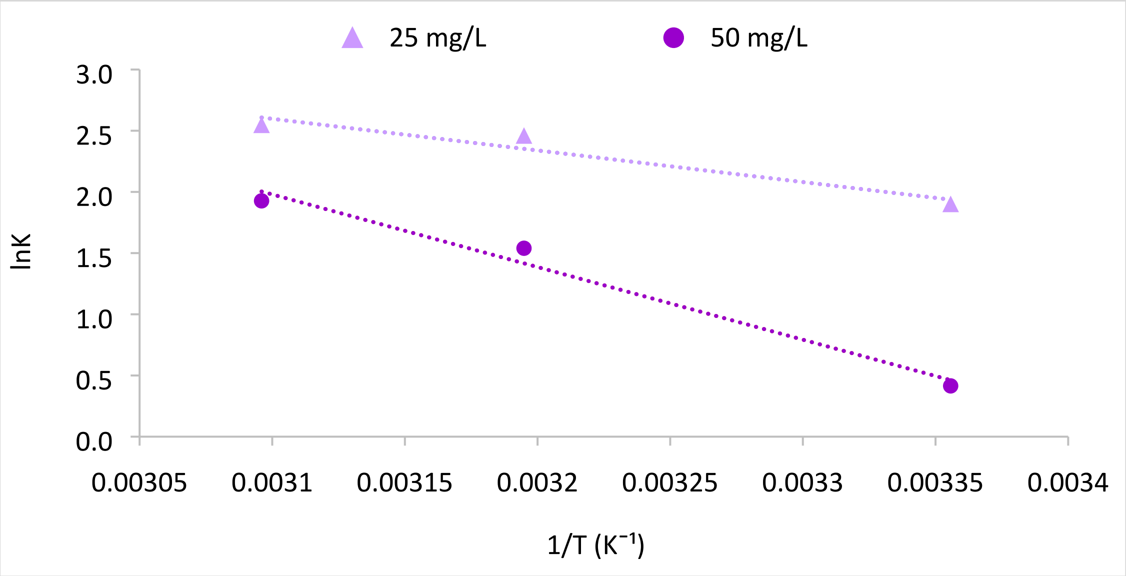

Figure S8: Thermodynamic plot for the adsorption of AV by biochar (Ci=25 and 50 mg/L, pH=3, m=2 g/L).

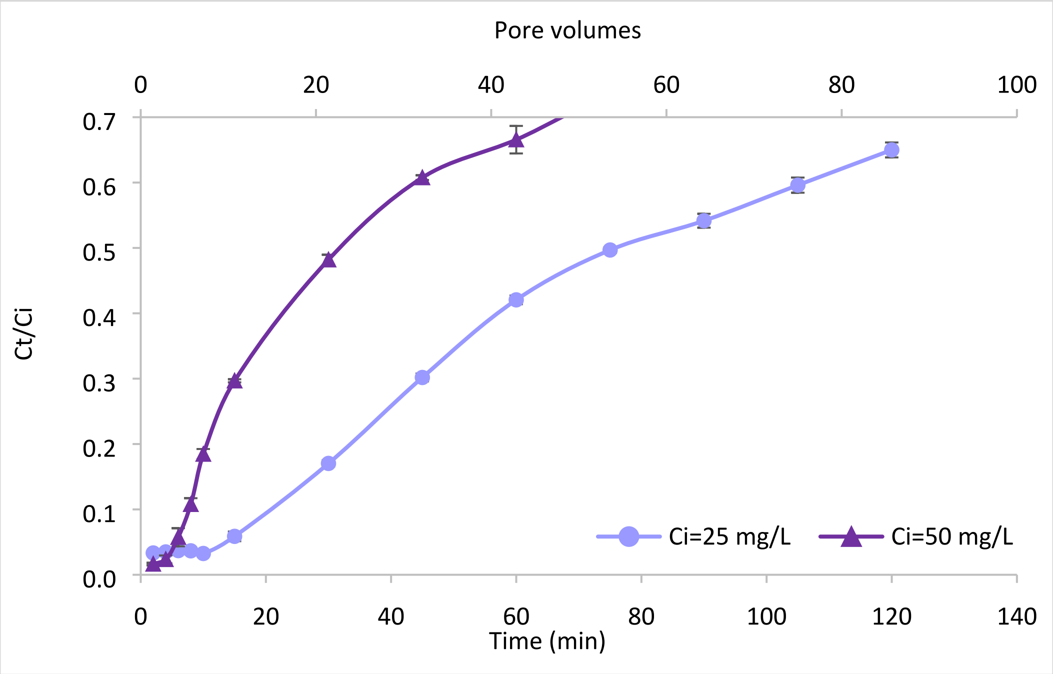

Figure S9: AV adsorption experiment in a fixed-bed column.

5. Responsive Surface Methodology (RSM) for dye removal by biochar:

Response surface methodology (RSM) is an effective tool for optimizing the process when a combination of several independent variables and their interactions affect desired responses[1,2]. In using the RSM approach, batch runs were conducted in CCD model designed experiments

to visualize the effects of independent factors on the response and the results along with the experimental conditions. A five-level CCD was performed to evaluate the influence of the 4 variables on the dye removal efficiency. Table S3 lists the process control variables and their limits. The center points are used to determine the experimental error and the reproducibility of the data. The independent variables are coded based on (−1, +1) interval where the low and high levels are coded as−1 and +1, respectively. Table S4 shows the experimental design points consisting of 2n factorial points with 2n axial points and Nc central points and the test results for the response variables. The axial points are located at a distance ofαfrom the center and make the design rotatable. In this study,α value was fixed at 2 (rotatable).

Table S3 Process control variables and their limits

**Factors**		**Levels**	
	**Low(-1)**	**Central (0)**	**High (+1)**
X_1, Intial dye concentration (mg/L)	25	37.5	50
X_2, pH	3	6	9
X_3, Dose of adsorbent (g/L)	0.5	1.25	2
X_4, Time (min)	60	150	240

This method is suitable for fitting a quadratic sur- face, and it helps to optimize the effective parameters with a minimum number of experiments and analyze the interaction between the parameters. Efficiency in order to evaluate the influence of operating parameters on the removal efficiency of AV, four main factors were chosen: Initial dye concentration (g/L) (X_1), pH (X_2), a dose of adsorbent (g/L)(X_3) and sorption time (min)(X_4).

Table S4 Data statistics of model variables

**Run**	**X_1**	**X_2**	**X_3**	**X_4**	**% Removal**
1	25.000	3.000	0.500	60.000	19.000
2	50.000	3.000	0.500	60.000	16.000
3	25.000	9.000	0.500	60.000	18.000
4	50.000	9.000	0.500	60.000	16.000
5	25.000	3.000	2.000	60.000	53.000

6	50.000	3.000	2.000	60.000	42.000
7	25.000	9.000	2.000	60.000	46.000
8	50.000	9.000	2.000	60.000	32.000
9	25.000	3.000	0.500	240.000	48.958
10	50.000	3.000	0.500	240.000	39.042
11	25.000	9.000	0.500	240.000	39.000
12	50.000	9.000	0.500	240.000	28.000
13	25.000	3.000	2.000	240.000	95.375
14	50.000	3.000	2.000	240.000	80.458
15	25.000	9.000	2.000	240.000	81.125
16	50.000	9.000	2.000	240.000	72.000
17	12.500	6.000	1.250	150.000	61.167
18	62.500	6.000	1.250	150.000	48.833
19	37.500	0.000	1.250	150.000	58.500
20	37.500	12.000	1.250	150.000	43.500
21	37.500	6.000	0.250	150.000	9.600
22	37.500	6.000	2.750	150.000	64.667
23	37.500	6.000	1.250	-30.000	42.000
24	37.500	6.000	1.250	330.000	81.667
25	37.500	6.000	1.250	150.000	82.000
26	37.500	6.000	1.250	150.000	82.000
27	37.500	6.000	1.250	150.000	82.000
28	37.500	6.000	1.250	150.000	82.000
29	37.500	6.000	1.250	150.000	82.000
30	37.500	6.000	1.250	150.000	82.000

The plot of the experimental removal (%) values versus those calculated from the equation indicated a good fit, as presented in Figure 1.

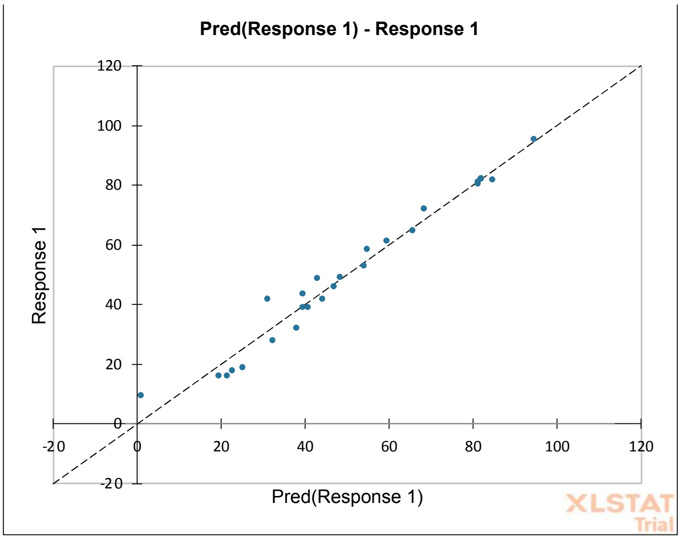

Figure S 10: The experimental data versus the predicted data of normalized removal of AV.

Table S5 ANOVA of the quadratic models for dye removal

Source	DF	Sum of squares	Mean squares	F	Pr > F
Model	14	18176.310	1298.308	43.026	**<0.0001**
X_1	1	413.548	413.548	13.705	**0.002**
X_2	1	350.434	350.434	11.613	**0.004**
X_3	1	6275.631	6275.631	207.976	**<0.0001**
X_4	1	4301.181	4301.181	142.542	**<0.0001**
X_1^2	1	645.579	645.579	21.395	**0.000**
$X_1 X_2$	1	0.458	0.458	0.015	0.904
$X_1 X_3$	1	33.423	33.423	1.108	0.309
$X_1 X_4$	1	13.984	13.984	0.463	0.506
X_2^2	1	1183.862	1183.862	39.234	**<0.0001**
$X_2 X_3$	1	19.599	19.599	0.650	0.433
$X_2 X_3$	1	41.307	41.307	1.369	0.260
X_3^2	1	3597.436	3597.436	119.220	**<0.0001**
$X_3 X_4$	1	305.886	305.886	10.137	**0.006**
X_4^2	1	993.982	993.982	32.941	**<0.0001**
Residual	15	452.621	30.175		

Table S5 shows the ANOVA results for the regression parameters of the predicted response surface quadratic model for the AV removal by the biochar. The mean sum of the squares was achieved by dividing the sum of the squares, model and error variance by the degree of freedom. F value for color removal by biochar is 43.02 and Prob>F was less than 0.0001, implying that model is relevant and significant. In this case, X_1, X_2, X_3, X_4, X_1^2, X_2^2, X_3^2, X_4^2,

X_3X_4 are significant model terms. Table S5 indicates that the biochar dose and sorption time are the most significant factors governing dye removal by biochar. The developed model suggests that initial dye concentration of 25 mg/L, pH3, 2g/L biochar and sorption time of 240 min are optimum conditions for maximum color removal of biochar.

Table S6 Adsorption of dyes bybiochar in ternary mixture

S.No	Dye	% Removal
1	AV	86
2	AG	70
3	MB	92

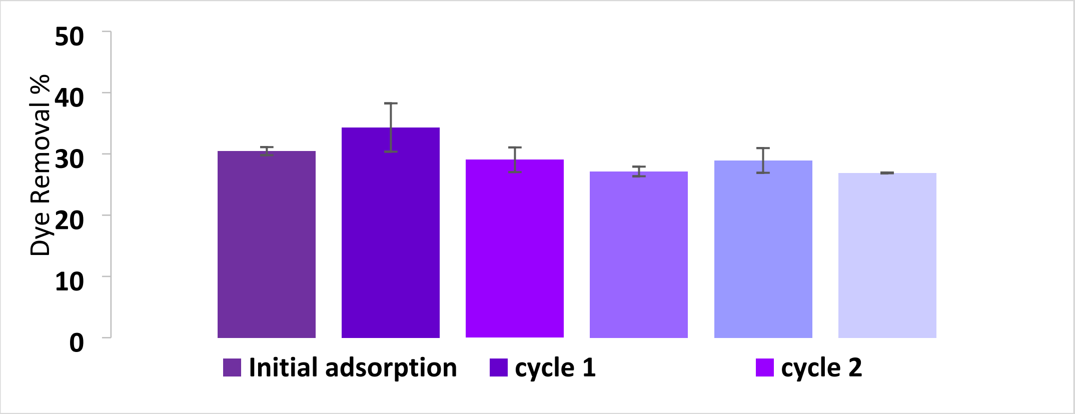

Figure S11: Removal of AV after 5 regeneration cycles of the biochar by 100% ethanol. Adsorption experiment after regeneration with following conditions: Ci=50 mg/L, pH=3, m=2 g/L, t=40 min, T=25°C.

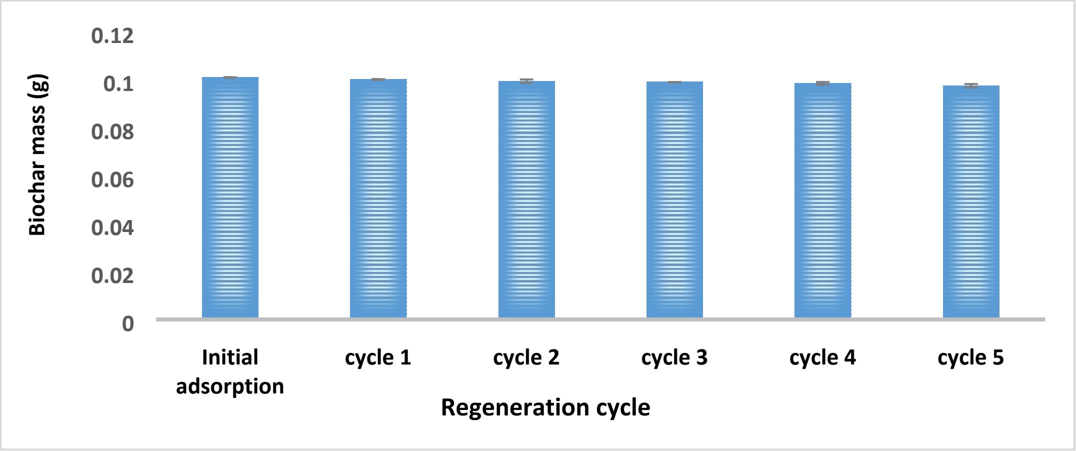

Figure S12: Biochar mass after each regeneration.

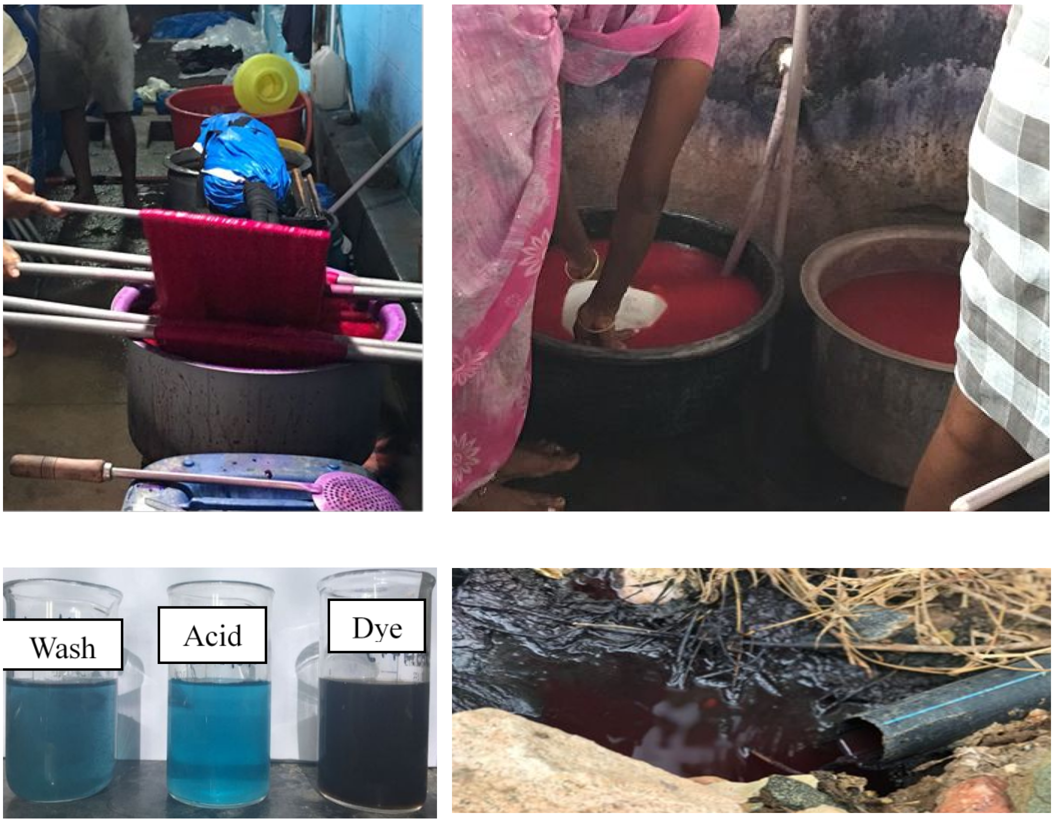

Figure S13: Real dyeing households in Southern India, three affluent types from the dyeing process, and wastewater discharged to the environment (effluents mixed).

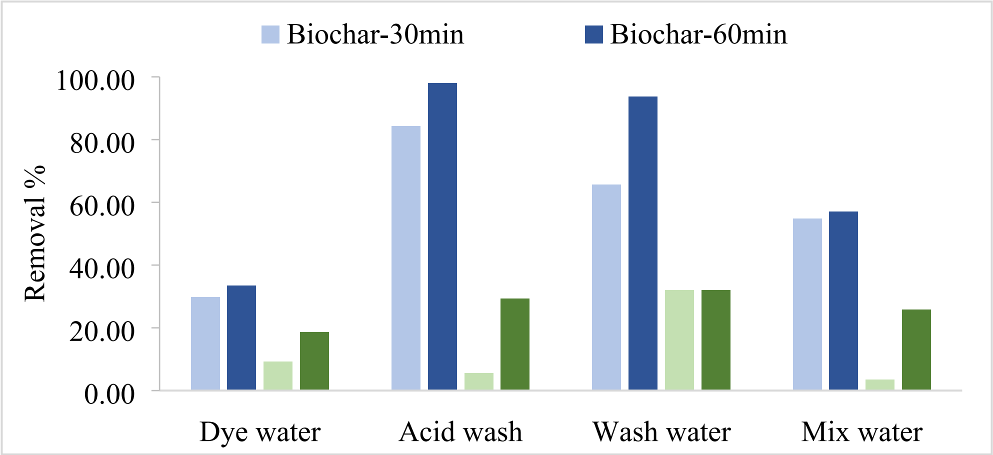

Figure S14: Adsorption experiment with real textile wastewater different effluents. Comparison between biochar and GAC after 30 min and 60 min of treatment.

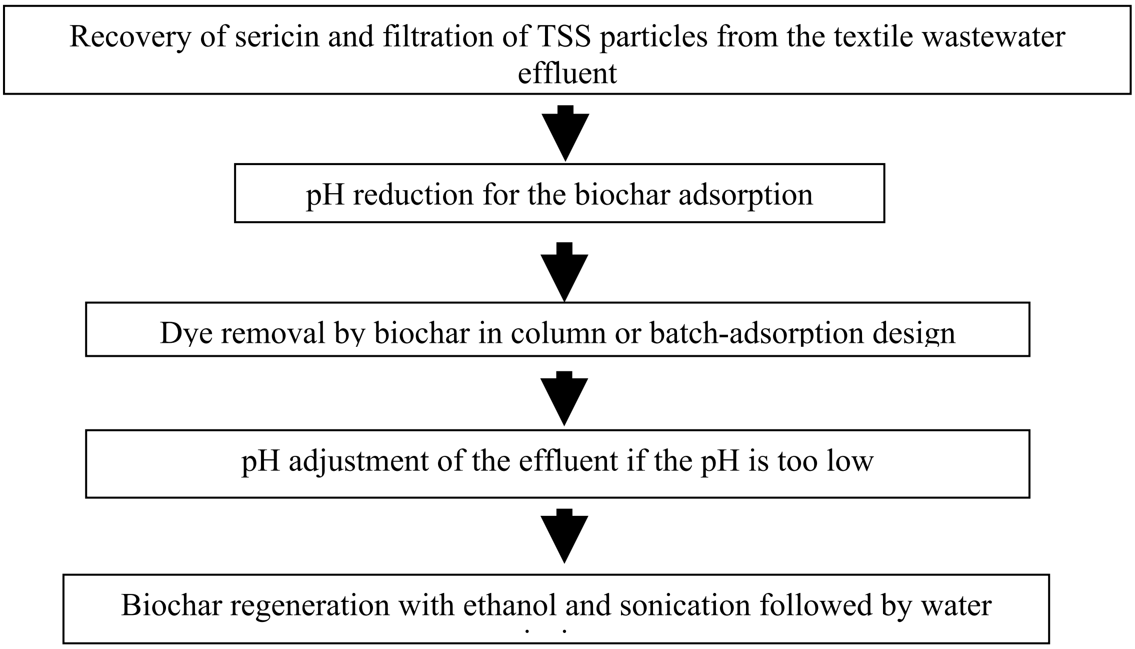

Figure S15: Schematic chart of the suggested textile dye wastewater adsorption system.

References

(1) Manikandan, P.; Palanisamy, P. N.; Baskar, R.; Sakthisharmila, P. Influence of Chemical Structure of Reactive Blue (Diazo), Direct Red (Diazo) and Acid Violet (Triaryl Alkane) Dyes on the Decolorization Efficiency by Photo Assisted Chemical Oxidation Process (PACO). *j. Eng.Technol.Research* **2017**, *5* (1), 01–14.

(2) Uddin, M. K. A Comparitive Study on Silk Dyeing. **2010**, No. December, 1–6.

(3) Wang, L.; Yan, W.; He, C.; Wen, H.; Cai, Z.; Wang, Z.; Chen, Z.; Liu, W. Microwave-Assisted Preparation of Nitrogen-Doped Biochars by Ammonium Acetate Activation for Adsorption of Acid Red 18. *Appl. Surf. Sci.* **2018**, *433*, 222–231. https://doi.org/10.1016/j.apsusc.2017.10.031.

(4) Umam, K.; Fitria, N. Surface Modification of Polyester Fiber with Sericin for Cold Reactive Dyeing. *AIP Conf. Proc.* **2018**, *2049* (December). https://doi.org/10.1063/1.5082406.

(5) Ghosh, S.; S. Rao, R.; Nambiar, K. S.; Haragannavar, V. C.; Augustine, D.; Sowmya, S. V. Sericin, a Dietary Additive: Mini Review. *J. Med. Radiol. Pathol. Surg.* **2019**, *6* (1), 4–8. https://doi.org/10.15713/ins.jmrps.153.

(6) Kan, C. W. Effect of Water Hardness on Acid Dyeing with Silk. *Autex Res. J.* **2008**, *8* (2), 54–56.

(7) Zhou, Y.; Zhang, J.; Tang, R. C.; Zhang, J. Simultaneous Dyeing and Functionalization of Silk with Three Natural Yellow Dyes. *Ind. Crops Prod.* **2015**, *64* (1), 224–232. https://doi.org/10.1016/j.indcrop.2014.09.041.

(8) Çapar, G.; Aygün, S. S. Characterization of Sericin Protein Recovered from Silk Wastewaters. *Turk Hij. ve Deney. Biyol. Derg.* **2015**, *72* (3), 219–234. https://doi.org/10.5505/TurkHijyen.2015.47113.

(9) Tong, X. J.; Li, J. Y.; Yuan, J. H.; Xu, R. K. Adsorption of Cu(II) by Biochars Generated

from Three Crop Straws. *Chem. Eng. J.* **2011**, *172* (2–3), 828–834. https://doi.org/10.1016/j.cej.2011.06.069.

(10) Yaroshchuk, A.; Ribitsch, V. Role of Channel Wall Conductance in the Determination of ζ-Potential from Electrokinetic Measurements. *Langmuir* **2002**, *18* (6), 2036–2038. https://doi.org/10.1021/la015557m.
